# Single-nucleus multiome shows motor neuron glutamate overactivation in amyotrophic lateral sclerosis

**DOI:** 10.1093/brain/awaf426

**Published:** 2025-11-11

**Authors:** Eriko Takeuchi, Yoshiaki Yasumizu, Junko Morita, Masakazu Ishikawa, Kotaro Ogawa, Daisuke Motooka, Daisuke Okuzaki, Miho Nagata, Yasuki Ishihara, Yohei Miyashita, Yoshihiro Asano, Kohji Mori, Eiichi Morii, Goichi Beck, Yuko Saito, Shigeo Murayama, Hideki Mochizuki, Seiichi Nagano

**Affiliations:** Department of Neurology, The University of Osaka Graduate School of Medicine, Suita, Osaka 565-0871, Japan; Department of Neurotherapeutics, The University of Osaka Graduate School of Medicine, Suita, Osaka 565-0871, Japan; Integrated Frontier Research for Medical Science Division, Institute for Open and Transdisciplinary Research Initiatives (OTRI), The University of Osaka, Suita, Osaka 565-0871, Japan; Department of Neurology, The University of Osaka Graduate School of Medicine, Suita, Osaka 565-0871, Japan; Integrated Frontier Research for Medical Science Division, Institute for Open and Transdisciplinary Research Initiatives (OTRI), The University of Osaka, Suita, Osaka 565-0871, Japan; Department of Experimental Immunology, Immunology Frontier Research Center, The University of Osaka, Suita, Osaka 565-0871, Japan; Department of Neurology, Yale School of Medicine, New Haven, CT 06510, USA; Department of Neurotherapeutics, The University of Osaka Graduate School of Medicine, Suita, Osaka 565-0871, Japan; Genome Information Research Center, The University of Osaka, Suita, Osaka 565-0871, Japan; Department of Neurology, The University of Osaka Graduate School of Medicine, Suita, Osaka 565-0871, Japan; Integrated Frontier Research for Medical Science Division, Institute for Open and Transdisciplinary Research Initiatives (OTRI), The University of Osaka, Suita, Osaka 565-0871, Japan; Genome Information Research Center, Research Institute for Microbial Diseases, The University of Osaka, Suita, Osaka 565-0871, Japan; Integrated Frontier Research for Medical Science Division, Institute for Open and Transdisciplinary Research Initiatives (OTRI), The University of Osaka, Suita, Osaka 565-0871, Japan; Genome Information Research Center, Research Institute for Microbial Diseases, The University of Osaka, Suita, Osaka 565-0871, Japan; Department of Cardiovascular Medicine (IRUD Analysis Center), The University of Osaka Graduate School of Medicine, Suita, Osaka 565-0871, Japan; Department of Cardiovascular Medicine (IRUD Analysis Center), The University of Osaka Graduate School of Medicine, Suita, Osaka 565-0871, Japan; NCVC Biobank, National Cerebral and Cardiovascular Center, Suita, Osaka 564-8565, Japan; Department of Cardiovascular Medicine (IRUD Analysis Center), The University of Osaka Graduate School of Medicine, Suita, Osaka 565-0871, Japan; Department of Genomic Medicine, National Cerebral and Cardiovascular Center, Suita, Osaka 564-8565, Japan; Department of Cardiovascular Medicine (IRUD Analysis Center), The University of Osaka Graduate School of Medicine, Suita, Osaka 565-0871, Japan; Department of Genomic Medicine, National Cerebral and Cardiovascular Center, Suita, Osaka 564-8565, Japan; Department of Psychiatry, The University of Osaka Graduate School of Medicine, Suita, Osaka 565-0871, Japan; Integrated Frontier Research for Medical Science Division, Institute for Open and Transdisciplinary Research Initiatives (OTRI), The University of Osaka, Suita, Osaka 565-0871, Japan; Department of Pathology, The University of Osaka Graduate School of Medicine, Suita, Osaka 565-0871, Japan; Department of Neurology, The University of Osaka Graduate School of Medicine, Suita, Osaka 565-0871, Japan; Department of Neuropathology (Brain Bank for Aging Research), Tokyo Metropolitan Geriatric Hospital and Institute of Gerontology, Itabashi, Tokyo 173-0015, Japan; Brain Bank for Neurodevelopmental, Molecular Research Center for Children’s Mental Development, Neurological and Psychiatric Disorders, The University of Osaka United Graduate School of Child Development, Suita, Osaka 565-0871, Japan; Department of Neurology, The University of Osaka Graduate School of Medicine, Suita, Osaka 565-0871, Japan; Integrated Frontier Research for Medical Science Division, Institute for Open and Transdisciplinary Research Initiatives (OTRI), The University of Osaka, Suita, Osaka 565-0871, Japan; Department of Neurology, National Hospital Organization Osaka Toneyama Medical Center, Toyonaka, Osaka 560-0871, Japan; Department of Neurology, The University of Osaka Graduate School of Medicine, Suita, Osaka 565-0871, Japan; Department of Neurotherapeutics, The University of Osaka Graduate School of Medicine, Suita, Osaka 565-0871, Japan; Integrated Frontier Research for Medical Science Division, Institute for Open and Transdisciplinary Research Initiatives (OTRI), The University of Osaka, Suita, Osaka 565-0871, Japan; Department of Neurodevelopmental and Neurodegenerative Disease Research, The University of Osaka United Graduate School of Child Development, Suita, Osaka 565-0871, Japan

**Keywords:** amyotrophic lateral sclerosis, single nucleus RNA-seq, single nucleus ATAC-seq, GWAS, spinal cord, motor cortex

## Abstract

Amyotrophic lateral sclerosis (ALS) is a neurodegenerative disease that causes motor neuron degeneration. However, the mechanisms underlying the selective vulnerability of motor neurons and the involvement of non-motor neuron cells in ALS remain unclear.

To investigate ALS pathology at the cellular level, we performed a single-nucleus multiome analysis, including RNA sequencing and chromatin accessibility profiling, on the motor cortex (75 583 nuclei) and spinal cord (62 711 nuclei) from patients with ALS (*n* = 6) and controls (*n* = 6).

Our results revealed significant gene expression changes specifically in spinal motor neurons, including upregulation of a metabotropic glutamate receptor, *GRM5*, and enhanced glutamate signalling. By integrating genome-wide association study data, we identified ALS-associated single nucleotide polymorphisms (SNPs) in regulatory regions, suggesting cell-type-specific enrichment of risk, especially in microglia.

These findings suggest that changes in spinal motor neurons and their surrounding environment, including glutamate signalling, may be involved in ALS pathology. The study also provides valuable resources for future research on the underlying mechanisms and potential therapeutic targets.

## Teaser

Takeuchi *et al.* used advanced single-cell analysis to examine brain and spinal cord tissues from patients with ALS. They found that spinal motor neurons show abnormal gene expression and enhanced glutamatergic signalling, offering new insights into ALS pathology and identifying potential therapeutic targets.

## Introduction

Amyotrophic lateral sclerosis (ALS) is a progressive neurodegenerative disease characterized by the selective degeneration of upper motor neurons in the cerebral cortex and lower motor neurons in the brainstem and spinal cord (SC). Further, in more than 95% of ALS cases, transactive response (TAR) DNA-binding protein 43 (TDP-43) is lost from the neuronal nuclei and forms abnormal aggregates in the cytoplasm.^1,2^ However, the mechanisms underlying the specific degeneration of the upper and lower motor neurons remain unclear.

Cell-autonomous mechanisms in motor neurons and non-cell-autonomous mechanisms in motor neurons and glial cells contribute to ALS pathology;^2,3^ however, the underlying mechanism remains poorly understood. ALS is now recognized as a complex disease involving interactions with surrounding cells rather than solely affecting motor neurons. Recent advances in single-nucleus RNA-sequencing (snRNA-seq) have facilitated studies on the brains of post-mortem patients with ALS and frontotemporal dementia, revealing the genetic vulnerability of cortical motor neurons,^4,5^ and the molecular identities of cortical motor and spindle neurons in the prefrontal cortex.^5^ Nonetheless, molecular changes in the lower motor neurons and comparisons with the upper motor neurons remain insufficiently explored.

Various approaches to investigate the genetic factors underlying ALS have been actively pursued.^6,7^ Genome-wide association studies (GWAS) have identified numerous common variants associated with ALS risk.^8,9^ Additionally, epigenetic changes, particularly the regulation of gene expression through chromatin accessibility, are found in ALS.^10,11^ Although these studies have significantly contributed to our understanding of ALS, few have integrated gene expression changes, chromatin accessibility and GWAS signals, highlighting the importance of such integrative approaches.

In this study, we applied multiome technology (snRNA-seq and assay for transposase-accessible chromatin using sequencing; ATAC-seq) to human cortical and SC samples for simultaneously analysing gene expression changes and chromatin accessibility at the single-nucleus level in sporadic ALS (sALS). Single-nucleus analyses of the SC are limited; therefore, we created a detailed cellular atlas for comparison with the brain. We then evaluated ALS-related genetic variations and cellular vulnerability through integrative analyses using GWAS hits and familial ALS-causative genes. We also identified cellular differences in the regulatory mechanisms of ALS risk genes, providing new insights into ALS pathology. This study provides a comprehensive understanding of ALS-related changes in the brain and SC and offers novel therapeutic targets for drug discovery. Furthermore, we provide a valuable resource for gene expression and epigenomic data from the motor cortex (MCX) and SC of the same patients.

## Materials and methods

### Human samples

We collected post-mortem tissue samples of the MCX and lumbar SC from post-mortem patients diagnosed with ALS (*n* = 6) based on the revised El Escorial and Airlie House criteria,^12^ with no familial history of the disease and confirmed pathological inclusions of TDP-43 in brain lesions, and from non-neurodegenerative disease controls (*n* = 6), whose samples were stored at −80°C at the National Center for Geriatrics and Gerontology and Department of Neurology, Graduate School of Medicine, Osaka University. The TDP-43 pathology stage determining ALS severity was defined according to the criteria of Brettschneider et al.^13^ Samples were frozen within 30 h post-mortem, and the RNA integrity number (RIN) was > 6.8. Samples were randomly and evenly assigned to either RNA-seq or multiome conditions across disease groups to reduce method-specific batch effects. Some samples were profiled multiple times by multiome and RNA-seq. For immunohistochemical analysis, lumbar SC tissues were isolated from patients with ALS (*n* = 3) and control individuals (*n* = 3), immediately fixed in paraformaldehyde and embedded in paraffin for sectioning. Ethical approval for this study was obtained from the Ethics Committee of Osaka University under the following approval IDs: 880-5 (human genome research), 03-086-025 (animal experiments) and 04838 (genetic experiments). Post-mortem tissue samples were obtained with consent for autopsy; however, written consent for research use was unavailable owing to the post-mortem interval. Instead, institutional ethical guidelines mandate public notification of archived tissue use, allowing families to inquire or opt out. Ethics Committee approval also covered the use of anonymized genetic data.

### Genomic DNA extraction and whole-genome sequencing

Genomic DNA was extracted from frozen brain tissue and subjected to whole-genome sequencing using the NovaSeq6000 platform (Illumina) with 150 base pair (bp) paired-end reads. Variant calling and annotation were performed using standard pipelines (FastQC, Trimmomatic, Burrows-Wheeler Aligner (BWA), Genome Analysis Toolkit (GATK) and ANNOVAR software). ALS-associated genes were screened based on the American College of Medical Genetics and Genomics (ACMG) guidelines,^14^ and no pathogenic variants were detected in known ALS risk genes. Detailed methods and parameters are provided in the Supplementary material, ‘Methods’ section.

### Nuclei isolation

Fresh-frozen MCX and lumbar SC tissues were processed for nuclei isolation using a modified density gradient centrifugation protocol based on the 10x Genomics and OptiPrep™ guidelines. Briefly, tissues were homogenized in lysis buffer, filtered and subjected to a three-layer iodixanol gradient. Nuclei were collected from the interphase and washed through serial centrifugation steps. All buffer compositions and centrifugation parameters are provided in the Supplementary material, ‘Methods’ section.

### Droplet-based single-cell sequencing

snRNA-seq and multiome libraries were prepared using the Chromium Next Gel Bead-In-Emulsion (GEM) Single Cell 3ʹ v.3.1 or Multiome ATAC + Gene Expression kits (10 × Genomics) and sequenced on a NovaSeq6000 platform (Illumina). Raw reads were processed using Cell Ranger (v.6.0.0) with intron-inclusive settings for RNA and Cell Ranger ARC for multiome data. Reference genomes and detailed parameters are provided in the Supplementary material, ‘Methods’ section.

### Pipeline for snRNA-seq analysis

snRNA-seq data were processed using Seurat (v.4.3.0).^15^ Low-quality cells and predicted doublets were excluded using DoubletFinder (v.2.0.3) with the established thresholds^16^ and demultiplexed with demuxlet.^17^ After SCTransform normalization and identification of highly variable genes (HVGs), dimensionality reduction was performed using principal component analysis (PCA) and uniform manifold approximation and projection (UMAP) with Harmony^18^ for batch correction. Clustering was conducted using the Leiden algorithm. For cell-type annotation, differentially expressed genes (DEGs) were identified using FindAllMarkers and matched to known marker genes. Five major cell types were initially defined in both the brain and SC. For fine clustering, subsets (e.g. neurons, astrocytes, microglia, oligodendrocytes) were reclustered separately, resulting in 42 subsets in the brain and 43 in the SC. Cell types were annotated with reference to the Azimuth brain atlas^15,19^ and published SC datasets.^20^ To assess changes in cell-type composition between ALS and controls, we applied scCODA (single-cell compositional data analysis),^21^ which uses Bayesian inference to estimate compositional shifts, reporting credibility scores and highest density intervals (HDIs). Functional enrichment analysis was performed using ClusterProfiler’s^22^ enrichGO function, which performs Gene Ontology (GO) enrichment, based on DEGs for each cluster. Further details, including parameter settings and quality control thresholds, are provided in the Supplementary material, ‘Methods’ section.

### Gene-level downstream analysis for the snRNA-seq dataset

We performed differential gene expression analysis between ALS (*n* = 6) and control (*n* = 6) samples using a pseudobulk strategy with DESeq2,^23^ incorporating age and sex as covariates. Genes with adjusted *P*-values (padj < 0.2) were considered significant. Functional enrichment of DEGs was assessed using enrichGO from the ClusterProfiler^22^ package. Additionally, we examined the expression patterns of 59 ALS-related genes^24-26^ and performed PCA followed by k-means clustering (k = 3) to classify cell-type-specific expression profiles. Detailed methods and parameter settings are provided in the Supplementary material, ‘Methods’ section.

### Cell–cell interaction analysis

We applied NeuronChat^27^ to assess changes in cell–cell communication (CCC) between ALS and control groups, using a neuron-specific ligand–target interaction database. The CCC strength was calculated as the product of ligand and target abundance between interacting cell types. For non-peptide neurotransmitters, the expression levels of synthetic enzymes and transporters were also incorporated in the computation of communication strength, as described in the original NeuronChat publication. Significant CCCs within the ALS and control groups were determined using permutation tests as implemented in NeuronChat (20 permutations), followed by multiple test correction, and considered links with an adjusted *P*-value (false discovery rate (FDR) < 0.05) to be significant. Receptors from NeuronChat were manually classified into biological families. For both SC and MCX, gene sets corresponding to each family were used to calculate module scores per cell using the AddModuleScore function in Seurat. Differences in module scores between ALS and control (HC) were evaluated within each cluster and family using Welch’s *t*-test, followed by FDR correction. Results were visualized as a dot plot. Details are provided in the Supplementary material, ‘Methods’ section.

### GWAS integration analysis with the snRNA-seq dataset

To identify ALS-associated cell populations, we applied the single-cell disease relevance score (scDRS)^28^ method to integrate snRNA-seq data with ALS GWAS summary statistics (GCST900271648).^8^ Gene weights were calculated using MAGMA (v.1.10),^29^ and the top 1000 disease-prioritized genes were used to score expression per cell. Cell-type level heritability enrichment was assessed by comparing scDRS scores against 1000 matched control sets. Additional GWAS datasets for multiple sclerosis, schizophrenia and height were used as controls. Reference genome annotations and population data were obtained from the 1000 Genomes Project and the MAGMA resource. Further details are provided in the Supplementary material, ‘Methods’ section.

### Spatial transcriptome data analysis

Spatial transcriptome data of the human SC were obtained from the ALS Spatial Transcriptomics database (https://als-st.nygenome.org/)^30^. Pre-annotated regions across tissue sections were aligned using affine transformation based on nearest-neighbour matching. Cell-type deconvolution was performed using Cell2location,^31^ guided by filtered scRNA-seq reference profiles. A regression model was trained to infer the spatial distribution of cell types in Visium samples. Full details of preprocessing, model parameters and alignment optimization are provided in the Supplementary material, ‘Methods’ section.

### Mutiome data and GWAS integration analysis

snATAC-seq data were processed using Signac^32^ after barcode-based label transfer from matched snRNA-seq profiles. Quality filtering was performed based on nucleosome and transcription start site (TSS) enrichment signals. Dimensionality reduction and integration were conducted using term frequency-inverse document frequency (TF-IDF), singular value decomposition (SVD) and Harmony.^18^ Cell-type-specific peaks were identified using MACS2^33^ and FindAllMarkers. To assess enrichment of ALS-associated single nucleotide polymorphisms (SNPs) in open chromatin regions (OCRs), we integrated fine-mapped GWAS SNPs (GCST900271648) with cell-type-specific peak sets. Linkage disequilibrium (LD) expansion, liftover and genomic annotation were conducted using PLINK (a whole-genome association analysis toolkit), the UCSC Genome Browser LiftOver tool, and ChIPseeker,^34,35^ respectively. Overlap enrichment was tested using *bedtools fisher*.^36^ To identify gene-regulatory candidate SNPs, peak–gene expression correlations were computed using LinkPeaks, and overlapping SNPs within OCRs were visualized using locusplotr.^37^ Full parameters and filtering criteria are described in the Supplementary material, ‘Methods’ section.

### Immunohistochemistry

Formalin-fixed paraffin-embedded (FFPE) human SC tissues were used to enable long-term preservation of rare post-mortem ALS samples. Lumbar cord (L5) samples were fixed with 10% buffered formalin, processed into paraffin blocks and sectioned at 4 μm thickness using a microtome as previously performed.^38,39^ For 3,3′-diaminobenzidine (DAB) staining, the sections were deparaffinized using Hemo-D, endogenous peroxidase was blocked with 3% H_2_O_2_ and antigen retrieval was performed by heating in a microwave for 15 min. Blocking was performed using 10% goat serum. The sections were incubated overnight at 4°C with a primary antibody against GRM5 (ab76316, diluted 1:300 in 2% normal goat serum). The following day, the sections were incubated with the secondary antibody, Dako EnVision + Single Reagent (HRP, Rabbit), for 1 h, followed by DAB staining for 5 min to visualize the target. The sections were counterstained with haematoxylin for 1 min, dehydrated using an alcohol series and cleared using Hemo-D.

### Histological quantification

The samples were observed using a SLIDEVIEW VS200 research slide scanner and Olympus software (OlyVIA) with a 20× objective lens. Ventral horn cells from ALS (*n* = 3) and control (*n* = 3) samples were identified, and only those with a visible nucleolus were included in the analysis. For each sample, two fields of view from the left and right sides were imaged, and the mean signal intensity was calculated for all ventral horn cells within the imaged area. The mean intensity of immunostaining was calculated using Fiji,^40^ following a previously described protocol^41^ to quantify DAB signal intensity. Briefly, DAB-stained images were separated using colour deconvolution and converted to black-and-white images, with the maximum threshold set to 225. All anterior horn cells visually identified within the field of view were set as regions of interest (ROIs). The mean signal intensity within each cell was calculated. A linear mixed model was used to examine the mean signal intensity between the ALS and control groups, with each patient considered a random effect. Specifically, a model was constructed using Python’s Statsmodels package *smf.mixedlm* function, with condition as the fixed effect and subject_id as the grouping variable.

### Animal experiments

All animals were maintained in accordance with the institutional guidelines of Osaka University. The technical protocols for animal experiments in this study were approved by the Small Animal Welfare Committee of Osaka University.

### Culture of cortical neurons

Neurons were cultured from the cerebral cortices of embryonic Day 15 (E15) C57BL/6 J mice, as previously described.^42^ Briefly, cerebral cortices were harvested from E15 C57BL/6 J mice, dissociated using papain (Worthington Biochemical Corp.), and seeded in 24-well plates (Greiner Bio-One) at a density of 5 × 10^5^ cells/well. The cells were initially cultured in Dulbecco’s Modified Eagle’s Medium (DMEM; Thermo Fisher Scientific) supplemented with 10% fetal bovine serum (FBS; HyClone) and 1× penicillin/streptomycin (Thermo Fisher Scientific) for 24 h. Subsequently, neurons were maintained in N1 medium (MACS; Miltenyi Biotec) containing 2% MACS NeuroBrew-21 (Miltenyi Biotec), 1 mM sodium pyruvate (Thermo Fisher Scientific), 1% Glutamax (Thermo Fisher Scientific), 27.5 μM 2-mercaptoethanol (Thermo Fisher Scientific) and 1× penicillin/streptomycin for 48 h. Finally, the medium was replaced with N1 medium supplemented with 2 μM cytosine β-D-arabinofuranoside (Ara-C; Sigma-Aldrich) for further culture.

### Preparation of viral vectors

Constructs of the lentiviral vector (pLKO.1) expressing control short hairpin RNA (shRNA) with no endogenous target genes (Cat. No. SHC002) and shRNA against the mouse TDP-43 gene (*TARDBP*) (Cat. No. TRCN0000174930; TDP-43 shRNA) were purchased from Sigma-Aldrich. Lentiviral production was performed as previously published.^42,43^ Briefly, 1.6 × 10^7^ 293FT cells (Thermo Fisher Scientific) were seeded in a 15-cm dish and cultured in DMEM supplemented with 10% FBS and 1× penicillin/streptomycin. At 6 h after plating, Δ8.9 (22.5 μg) and VSV-G (10 μg) plasmids were co-transfected with the shRNA construct (30 μg) using the CalPhos Mammalian Transfection Kit (Cat. No. 631 312). The medium containing the lentivirus was collected at 68 and 92 h post-transfection. The collected medium was subjected to ultracentrifugation at 44 396*g* for 140 min, and the pellet was resuspended in DMEM supplemented with 10% FBS and 1× penicillin/streptomycin to prepare the virus solution. For transduction, the viral solution was applied to cortical neurons at 1 day *in vitro* (DIV) for 6 h.

### Immunoblot analysis

Relative quantification of western blots was performed using Fiji.^40^ Glyceraldehyde-3-phosphate dehydrogenase (GAPDH) was used as a loading control. TDP-43 and GRM5 protein signals were normalized to their background signals and calculated relative to the GAPDH signal. The statistical significance of the difference in signal ratios between control shRNA and TDP-43 shRNA was evaluated using Dunnett’s multiple comparison test, which revealed a significant difference in GRM5 expression between the TDP-43 and control shRNA groups (*P* < 0.05).

## Results

### Single-nucleus transcriptional profiling of the motor cortex and spinal cord

**Figure 1:**
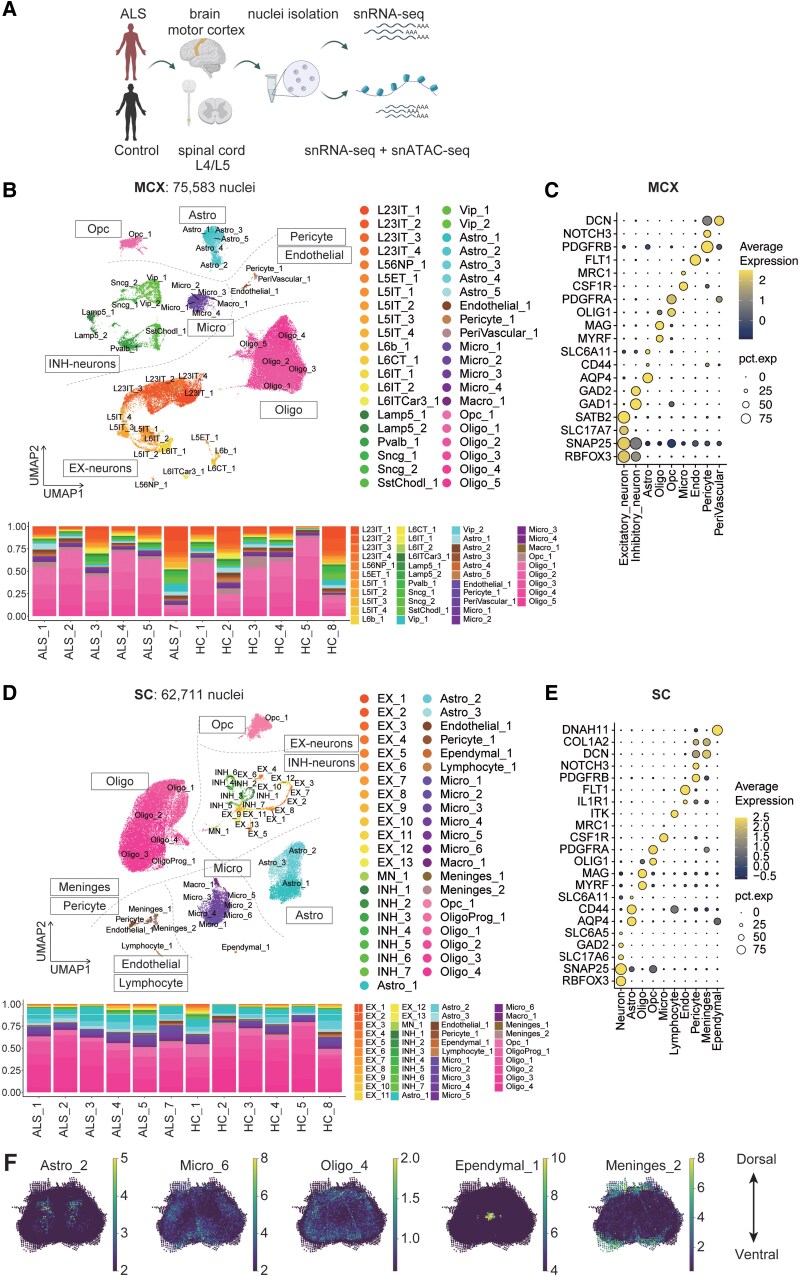
**Overview of the single-nucleus RNA sequencing of the motor cortex and spinal cord from patients with amyotrophic lateral sclerosis.** (**A**) Experimental design of single-nucleus RNA sequencing (snRNA-seq) and snMultiome-seq. Created in BioRender. Takeuchi, E. (2023) https://BioRender.com/z53sus7 (**B–E**) snRNA-seq analysis of motor cortex (MCX) (**B** and **C**) and spinal cord (SC) (**D** and **E**) samples. (**B** and **D**) Uniform manifold approximation and projection (UMAP) plot for cells with annotated clusters (detailed clusters designated as ThirdAnnotation) (*top*) and cell composition per sample (*bottom*). (**C** and **E**) Dot plots showing canonical marker genes for each cell type. (**F**) Spatial distribution of SC clusters. Spatial transcriptome data of the SC from a patient with amyotrophic lateral sclerosis (ALS)^30^ with predefined anatomical regions were used. Multiple sections from the SC were overlaid based on the anatomical clusters. Cell frequencies were inferred using Cell2location.^31^ Only representative cell types are illustrated; for all cell types, refer to Supplementary Fig. 2.

To capture the cell-type-specific gene expression changes involved in ALS pathology, we performed omics profiling using single nuclei. Clinically, ALS is characterized by impairments in both the upper motor neurons descending from the primary MCX and spinal lower motor neurons. Therefore, we focused on the MCX and SC. We obtained frozen tissue samples from the MCX (Brodmann area 4; MA4) and lumbar SC [the fourth and fifth lumbar (L4/5) segments] from six patients diagnosed with sALS confirmed by TDP-43 pathology and from six controls with no history of neurodegenerative diseases. We extracted nuclei and conducted snRNA-seq and multiome (RNA + ATAC) sequencing (Fig. 1A, Table 1 and Supplementary Table 1). After removing low-quality cells, we obtained data for 75 583 nuclei from the MCX and 62 711 nuclei from the SC (Supplementary Fig. 1A–C). We identified five major clusters—neurons, astrocytes, microglia, oligodendrocytes and others (vascular cells and lymphocytes)—using known marker genes in both the MCX and SC. Further clustering and annotation were performed for each major population by examining the expression of known marker genes and referencing annotations from integrated public data.^19,20^ We identified 42 clusters in the MCX and 43 clusters in the SC (Fig. 1B–E and Supplementary Fig. 1D). Additionally, for SC, we validated the spatial distribution of defined clusters using the spatial transcriptome profile of human SC tissue^30^ with a cell deconvolution algorithm (Fig. 1F and Supplementary Fig. 2A and B).

**Table 1:** Number of samples profiled in snRNA-seq and snRNA + ATAC-seq analysis

Tissue	snRNA-seq	snRNA + ATAC-seq	Total
**Brain (motor cortex)**			
CTRL	2	4	6
ALS	2	4	6
**Spinal cord (L4/5)**			
CTRL	2	4	6
ALS	2	4	6

The number of samples from control (CTRL) and amyotrophic lateral sclerosis (ALS) donors used for single-nucleus RNA sequencing (snRNA-seq) and single-nucleus multiome sequencing (snRNA + assay for transposase-accessible chromatin sequencing; ATAC-seq) analyses. Samples were obtained from motor cortex and spinal cord (L4/5) regions.

We identified 23 and 21 neuronal cell subpopulations in the MCX and SC groups, respectively. In the MCX, we defined 15 excitatory glutamatergic subpopulations characterized by the expression of *SLC17A7* and *SATB2* and eight inhibitory GABAergic subpopulations expressing *GAD1* and *GAD2*. These excitatory glutamatergic subpopulations include the extra-elencephalic projection neurons in layer 5 (L5ET_1; marker genes: *VAT1L*, *EYA4* and *THSD4*), which encompass motor neurons, also known as Betz cells, that directly control motor neurons and are affected in ALS. Among the inhibitory neurons, we identified parvalbumin-positive cells (Pvalb_1; *PVALB*), LAMP5 (lysosome-associated membrane glycoprotein 5)-positive cells, including rosehip (Lamp5_1, Lamp5_2), VIP (vasoactive intestinal peptide)-positive (Vip_1, Vip_2), somatostatin-positive (SstChodl_1) and *CCK*-positive cells (Sncg_1, Sncg_2). The characteristics of these major subtypes were consistent with those in previous reports.^5^ In the SC, we identified motor neurons (MN_1, *SLC5A7* and *ACLY*), which undergo cell death in ALS, along with 13 glutamatergic subpopulations expressing *SLC17A6* (EX_1–EX_13) and 7 GABA/glycinergic subpopulations expressing *GAD1*, *GAD2* and *SLC6A5* (INH_1–INH_7).

Among the non-neuronal cells in the MCX, the astrocyte group included subpopulations corresponding to fibrillary (high expression of *CD44* and *GFAP*) and protoplasmic astrocytes (low expression of *CD44* and *GFAP*). The microglial group comprised four microglial subpopulations (Micro_1, *SYNDIG1*, *NAV3*; Micro_2, *DOCK4*, *HIF1A*; Micro_3, *FTL*, *RPS11*; Micro_4, *NEFL* and *SCN1A*) and one macrophage subpopulation (Macro_1, *CSF1R* and *MRC1*). The oligodendrocyte group included five oligodendrocyte subpopulations and one oligo precursor cell (OPC) (*PDGFRA* and *OLIG1*). In the Others group, we identified endothelial cells (Endothelial_1, *FLT1* and *ABCB1*), perivascular fibroblasts (PeriVascular_1, *LAMA2* and *ABCA9*) and pericytes (Pericyte_1, *DLC1* and *PDGFRB*).

In the SC, the Astrocyte group included fibrillary astrocytes localized in the white matter (Astro_1; *CD44*, *CCDC85A*) and two protoplasmic astrocyte subpopulations (Astro_2, *SLC1A2*, *TENM2*; Astro_3, *GABRB1*, *GRID2*) localized in the grey matter, with Astro_3 also partially expressed in the white matter. The Microglia group comprised six microglial subpopulations (Micro_1: *KCNIP1* and *FOXP2*; Micro_2: *SPP1* and *IPCEF1*; Micro_3: *GPNMB* and *IQGAP2*; Micro_4: *SLC24A2* and *IL1RAPL1*; Micro_5: *GNA13* and *NAMPT*; Micro_6: *FTL* and *EEF1A1*) and one macrophage subpopulation (Macro_1: *MRC1* and *CD163*). The Oligodendrocyte group included four oligodendrocyte subpopulations (Oligo_1: *CCSER1* and *CACNA1B*; Oligo_2: *GNA14* and *COL18A1*; Oligo_3: *NLGN1* and *PLXDC2*; Oligo_4: *SGCZ* and *KCNMB4*), an OPC (Opc_1: *VCAN* and *PDGFRA*) and a related oligo progenitor cell (Oligoprog_1: *SEMA5B* and *GPR17*) that exhibited characteristics intermediate between OPCs and oligodendrocytes. In the Others group, we identified pericytes (Pericyte_1; *PDGFRB*), which likely included vascular smooth muscle cells (*MYH11*), putative meningeal fibroblasts (Meninges_1; *SLC4A4*), perivascular fibroblasts (Meninges_2; *DCN*, *ABCA8*), ependymal cells (Ependymal_1; *DNAH11*, *CFAP44*), lymphocytes (Lymphocyte_1; *CD96*, *ITK*) and endothelial cells (Endothelial_1; *FLT1*, *VWF*).

In terms of cell population frequency, oligodendrocytes were the most abundant in both the MCX (56.7 ± 23.3%) and SC (62.7 ± 10.3%), consistent with previous reports.^19,20^ In the MCX, this was followed by neurons (31.4 ± 21.5%) and astrocytes (7 ± 4.6%), whereas in the SC, astrocytes (17.6 ± 4.9%) and microglia (10.3 ± 3.7%) were the next most abundant (Fig. 1B and D). We constructed a detailed single-nucleus gene expression atlas derived from the MCX and lumbar SC of the corresponding donors, providing a foundational reference for capturing disease-specific cellular changes.

### Reduced oligodendrocyte subpopulation stabilizes synapses and protects motor neurons in ALS

**Figure 2:**
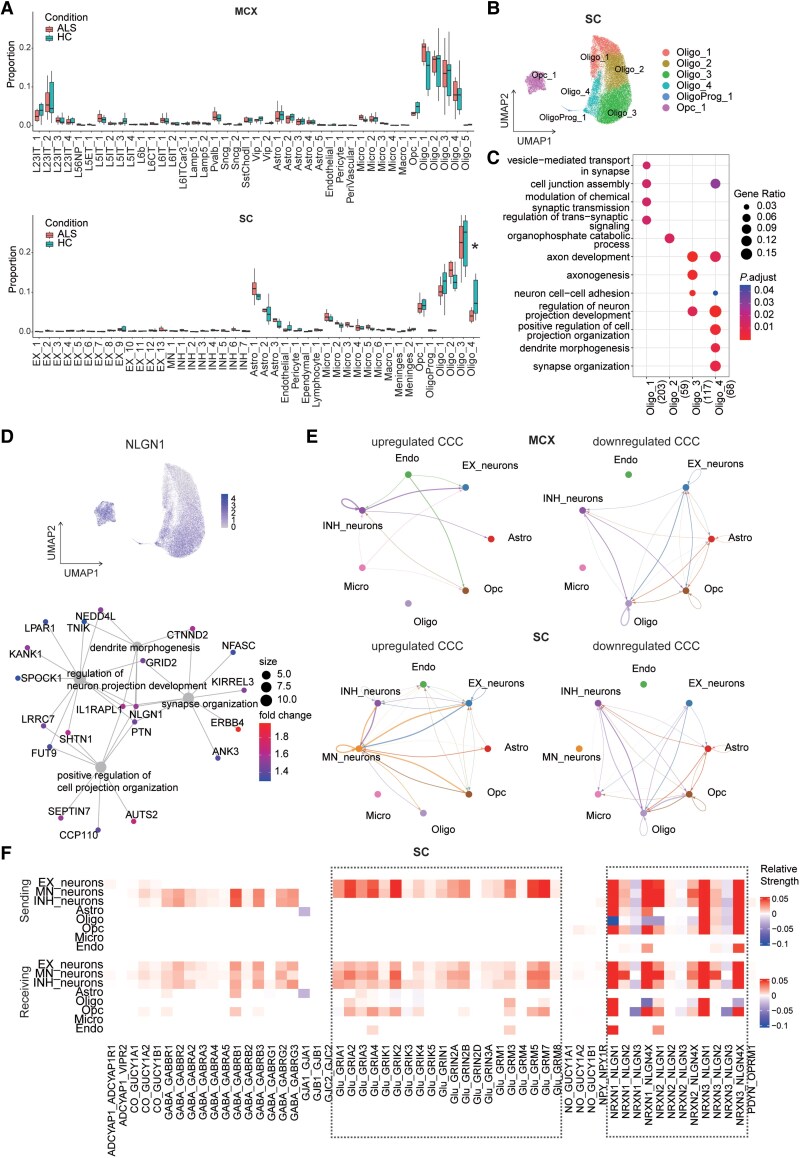
**Cell number and cell–cell interaction changes in patients with amyotrophic lateral sclerosis.** (**A**) Box plots showing the cell proportions in each cell cluster of each tissue. *Top*: Motor cortex (MCX; 42 clusters). *Bottom*: Spinal cord (SC; 43 clusters). Statistical analysis was performed using single-cell compositional data analysis (scCODA).^21^ *Cell clusters with credible effects (FDR < 0.1)^21^ (Supplementary Table 2). (**B**) UMAP plot shows the distribution of oligodendrocytes and oligodendrocyte precursor cells (OPCs) in the SC. (**C** and **D**) Characteristic pathways in oligodendrocytes. (**C**) Gene ontology (GO) Biological Process pathways enriched in differentially expressed genes (DEGs) from each cell cluster (Oligo_1–4). (**D**) FeaturePlot showing *NLGN1* expression, which is upregulated in Oligo_4, within all oligodendrocyte clusters (*top*). Enrichment map visualizing the genes involved in Oligo_4-enriched pathways (*bottom*). (**E** and **F**) Cell–cell communication (CCC) changes were analysed using NeuronChat.^27^ (**E**) Circle plot illustrating CCC changes in amyotrophic lateral sclerosis (ALS). The edge width represents the absolute difference in CCC intensity between the ALS and control samples. *Top*: MCX; *bottom*: SC. *Left*: Increased CCCs in ALS; *right*: decreased CCCs. (**F**) Heat map showing differences in signalling intensity between ALS and control samples for both sending (*top*) and receiving (*bottom*) signals in the SC. For each cell group and interaction pair, the sending and receiving signalling intensity is defined as the total communication strength from or to that cell type. The colour indicates the difference in signalling intensity between the ALS and control groups. FDR = false discovery rate; UMAP = uniform manifold approximation and projection.

To identify the vulnerable cell populations in ALS, we examined the quantitative and qualitative changes between ALS and control samples and the cellular localization of known pathogenic mutations associated with ALS. We first analysed changes in cell type composition using scCODA.^21^ In the MCX, no credible differences were observed between ALS and control samples for any cell type (Fig. 2A and Supplementary Table 2). However, in the SC, one oligodendrocyte subpopulation, Oligo_4, showed a reduction in patients with ALS (Fig. 2A). The SC oligodendrocyte clusters were classified into six detailed types: Oligodendrocyte (Oligo_1–4), Oligo-precursor cell (Opc_1) and related Oligo Progenitor cell (OligoProg_1) (Figs 1D and 2B and Supplementary Table 2). Within oligodendrocyte clusters, Oligo_4 exhibited higher expression of genes associated with synapse formation and stabilization (Fig. 2C). At the gene level, Oligo_4 specifically expressed pleiotrophin (*PTN*), which exerts neuroprotective effects on motor neurons,^44^ and neuroligin-1 (*NLGN1*), which binds to neurexins (*NRXN1–3*) and is involved in synapse stabilization and function (Fig. 2D). These findings suggest that changes in the composition of spinal oligodendrocytes, specifically those involved in synaptic regulation, are associated with ALS pathology.

### Enhanced glutamate signalling and altered intercellular communication in ALS motor neurons

Next, to capture changes in intercellular interactions in ALS, we performed cell–cell communication (CCC) analysis using NeuronChat,^27^ which focuses on neuron-specific ligand–receptor pairs based on gene expression data from each cell type. We subgrouped the detailed cell classifications (42 subpopulations in the MCX and 43 in the SC) into the following major categories: excitatory neurons, inhibitory neurons, motor neurons (in the SC), oligodendrocytes, OPCs, astrocytes, microglia and vascular cells (referred to as endo), which included endothelial cells and pericytes, as well as perivascular fibroblasts in the MCX, and ependymal and meningeal cells in the SC. We then compared the CCC networks of ALS and control samples. The total number of significant CCCs indicated no substantial change in the number of interactions between the ALS and control samples, with 258 and 254 CCCs in the MCX and 497 and 511 CCCs in the SC, respectively (Supplementary Fig. 3A). However, the intensity of CCC decreased in the MCX of patients with ALS (11.022) compared with that in the controls (11.844), whereas it increased in the SC of patients with ALS (19.934) compared with that in the controls (15.488) (Supplementary Fig. 3B). These results showed a contrasting change in CCC strength, with a decrease in MCX and an increase in SC.

A comparison of patients with ALS to controls across different cell types revealed a marked increase in CCC intensity between motor neurons and all other neurons and glia (astrocytes, OPCs and oligodendrocytes) in the SC (Fig. 2E). In contrast, only slight increases in communication intensity were observed in the MCX, primarily between the endo and inhibitory neurons, with some neurons and glia (Fig. 2E). In both the MCX and SC, the intensity of communication between oligodendrocytes and astrocytes with neurons other than motor neurons was reduced (Fig. 2E). Furthermore, when comparing patients with ALS with controls regarding the communication intensity of each ligand–receptor pair, two significant alterations were observed. First, in the SC, increased glutamate signalling and increased metabotropic glutamate receptors were observed in motor neurons (Fig. 2F and Supplementary Fig. 3D). This enhancement of glutamate signalling supports its critical role in ALS pathology, particularly in contributing to excitotoxicity.^45-47^ Second, Neuroligin-1 (*NLGN1*) signals mediated by neurexins (*NRXN1*-*NLGN1*, *NRXN2*-*NLGN1* and *NRXN3*-*NLGN1*) between neurons, OPCs, oligodendrocytes and other neurons were upregulated in the SC (Fig. 2F), whereas the overall neuroligin receptor expression in SC and MCX, and *NRXN1*-*NLGN1* signalling in MCX was decreased in these cell types (Supplementary Fig. 3C–E). *NLGN1* regulates the expression and function of glutamate receptors,^48^ and the dysregulation of neuroligin signalling implicates glutamate excitotoxicity in ALS. Overall, these findings highlight increased interactions in the SC and enhanced glutamate signalling, especially involving motor neurons in ALS.

### Distinct cell-type specificity in ALS-related gene expression and variant susceptibility

**Figure 3:**
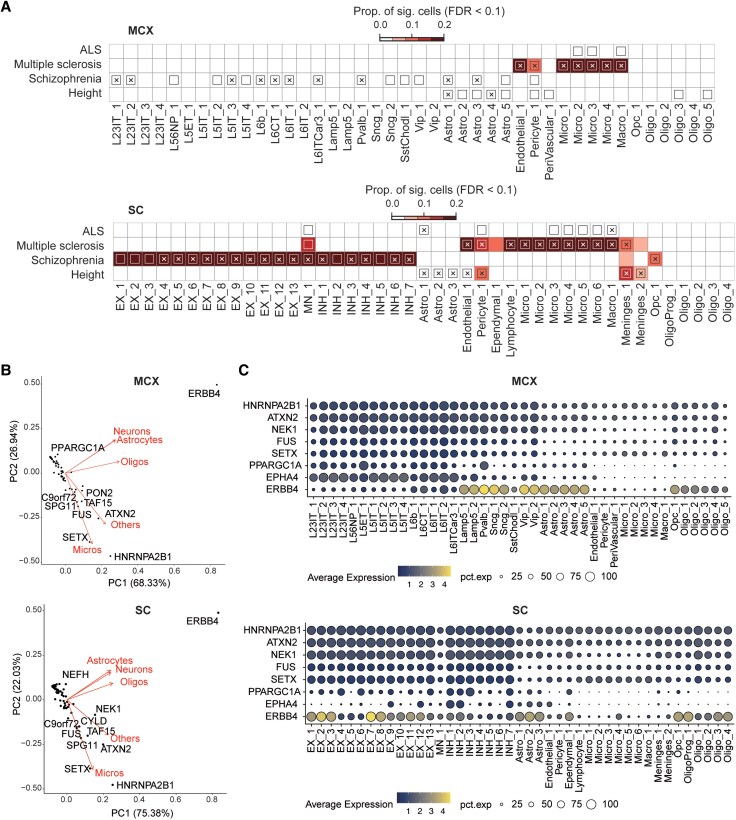
**Cell-type-specific enrichment of amyotrophic lateral sclerosis-related genes harbouring common and rare variants.** (**A**) Heat maps showing disease associations in the motor cortex (MCX; *top*) and spinal cord (SC; *bottom*), analysed using the single-cell disease relevance score (scDRS).^28^ As controls, we included multiple sclerosis (MS), schizophrenia (SCZ) and height. Heat map colours depict the proportion of significant cells (FDR < 0.1). Squares denote significant disease associations (FDR < 0.05), and cross symbols denote significant heterogeneity in association (FDR < 0.05). (**B**) Principal component analysis (PCA) biplots visualizing the expression patterns of 59 amyotrophic lateral sclerosis (ALS)-related genes in the MCX (*top*) and SC (*bottom*). PCs were calculated based on cell-type-specific gene expression. Red arrows indicate the loading vectors for each cell type. The direction represents the axis of maximal correlation with the expression of ALS-related genes, and the arrow length reflects the contribution of the cell type to the principal components. (**C**) Dot plots showing the expression of 59 manually curated genes harbouring ALS-associated rare variants for each cell type in the MCX (*top*) and the SC (*bottom*). The colour bar shows average gene expression levels within each cell type, and dot size represents the percentage of cells expressing each gene. FDR = false discovery rate.

We constructed a cellular atlas containing cell-population-specific gene expression information and used this resource to investigate the relationship between known ALS-related genes and cell types. As ALS involves both common and rare variants in the disease onset,^7,26^ we focused on each of these variants to explore potential cell-type specificity. First, we examined the polygenic risk of diseases associated with common variants. Using the scDRS,^28^ we assessed the polygenic disease risk at the single-cell level. The results showed that in the MCX, susceptibility was concentrated in microglia and macrophages, whereas in the SC, susceptibility was observed in microglia, macrophages, lymphocytes, endothelial cells, pericytes, astrocytes and motor neurons (Fig. 3A and Supplementary Fig. 4). The accumulation of susceptibility in microglia, endothelial cells, pericytes and astrocytes was consistent with previous findings.^49^ Additionally, accumulation of polygenic disease risk was observed in motor neurons.

Next, we investigated the cell specificity of genes harbouring rare variants associated with ALS using snRNA-seq data. We curated 59 genes reported to be associated with ALS^24-26^ and examined their expression patterns in different cell types. Accordingly, we performed PCA using their average expression across five major cell types (neurons, astrocytes, oligodendrocytes, microglia and others) in the MCX and SC (Fig. 3B). PCA revealed that ALS-related rare variants could be grouped into three distinct patterns. The first group comprised genes with ubiquitous expression across different cell types. The second group, including *ATXN2, SETX, FUS* and *TAF15*, was highly expressed in microglia and other cell types, including endothelial cells, pericytes and perivascular fibroblasts in the MCX, as well as in ependymal and meningeal cells in the SC. Finally, the third group exhibited a unique pattern, as exemplified by *ERBB4* (Fig. 3B and C and Supplementary Fig. 5A–D). *ERBB4* was highly expressed in neurons, astrocytes and oligodendrocytes, with predominant expression in inhibitory neurons in the MCX and excitatory neurons in the SC (Fig. 3B and C). By integrating our extensive resources with the established genetic information, we found that rare and common variants contribute differently to the genetic burden in ALS, underscoring the complexity of the genetic predisposition of the disease.

### Gene expression changes are the most pronounced in spinal motor neurons

**Figure 4:**
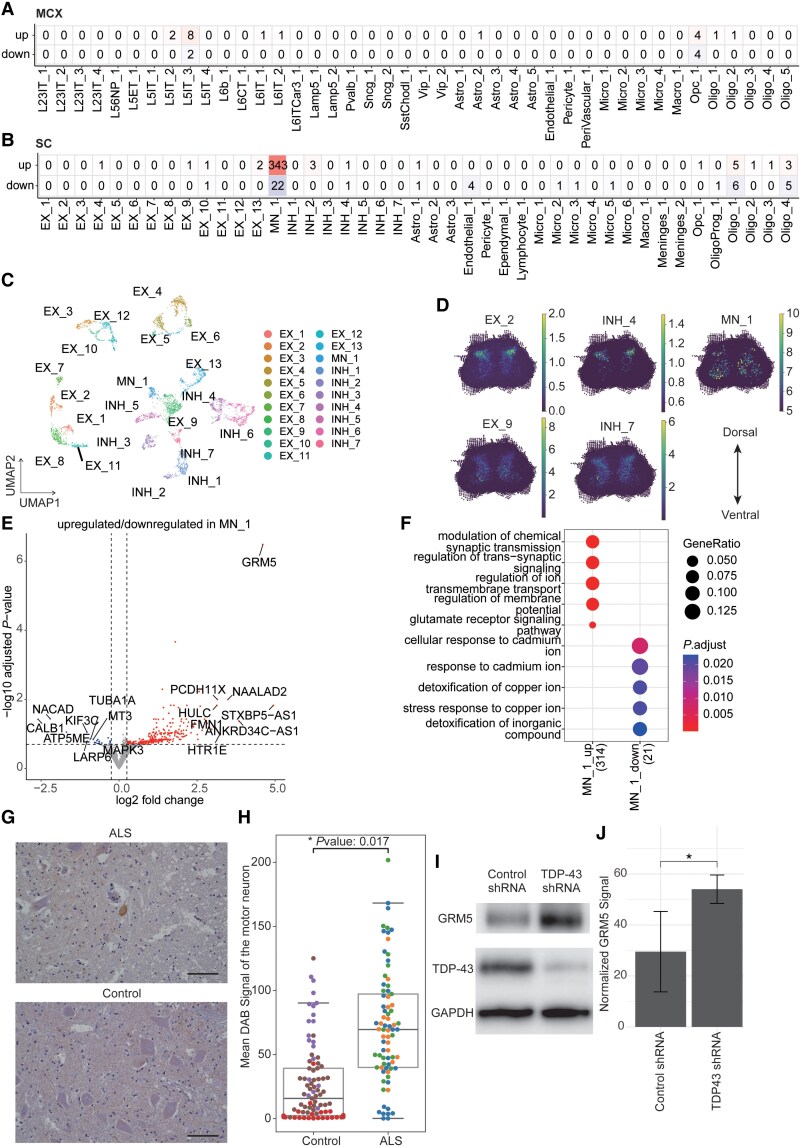
**Amyotrophic lateral sclerosis-associated gene expression changes in motor neurons.** (**A** and **B**) Heat maps showing the number of upregulated genes and downregulated genes in amyotrophic lateral sclerosis (ALS) per cluster in the motor cortex (MCX) (**A**) and spinal cord (SC) (**B**). The numbers in the boxes and colours indicate the number of differentially expressed genes (DEGs). Statistically significant genes were defined as FDR < 0.2, both in the MCX and SC. (**C**) UMAP plots showing subclusters of neurons in the SC. (**D**) Spatial transcriptome data of SC from human patients with ALS^30^ and cellular proportions predicted using Cell2location.^31^ (**E**) Volcano plot showing gene expression changes in MN_1. Upregulated genes are coloured in red, and downregulated genes are coloured in blue. Dashed lines indicate the applied cut-offs (horizontal: adjusted *P*-value = 0.2; vertical: log_2_ fold-change = ±0.25). (**F**) Dot plot shows the characteristic pathways within the upregulated or downregulated genes in MN_1. (**G**) Immunohistochemistry showing that GRM5 expression is elevated in spinal ventral horn cells (motor neurons) in ALS compared with that in the control (representative images). The DAB (3,3′-diaminobenzidine) signal is represented by a brown colour. (**H**) Swarm plot shows the mean DAB signals in the ventral horn within each sample. The colours in the plot represent individual samples. Statistical analysis was performed using a linear mixed model in the statsmodels package. (**I**) Protein expression levels of GRM5, TDP-43 and GAPDH. (**J**) Expression levels of each protein against those of GAPDH were quantified (*n* = 3 in each group). The source data are in the Supplementary material. FDR = false discovery rate; UMAP = uniform manifold approximation and projection.

We then performed a pseudo-bulk differential gene expression analysis to capture these changes in ALS at the cell-type level. In the MCX, genes were upregulated in subtypes of excitatory neurons (L5IT_3, DEGs: *WWC1*, *KCNMB2*, *MAOB*; L5IT_2, *GULP1*), oligodendrocytes (Oligo_1, DEGs: *CPS1*) and OPCs (Opc_1, DEGs: *FAM162A*, *CNTNAP4*). Further, several downregulated genes were detected in the excitatory neurons (L5IT_3, DEGs: *ADAMTSL1*) and OPCs (Opc_1, DEGs: *SMOC1, CHST8*) (Fig. 4A and Supplementary Fig. 6A). No DEGs were detected in L5ET_1, which corresponded to Betz cells. In contrast, the spinal motor neurons (MN_1) exhibited the highest number of DEGs, with 343 upregulated and 22 downregulated. Other cell types with notable changes included oligodendrocytes (Oligo_1, DEGs: *TTC21A*, *ZNF100*; Oligo_4, DEGs: *PPFIBP2*, *ZNF100*), which showed upregulated genes, and endothelial cells (Endothelial_1, DEGs: *KRT16*, *TBX15*), which showed downregulated genes (Fig. 4B and Supplementary Fig. 6B).

### ALS-specific glutamate signalling changes in the spinal motor neurons

To investigate the ALS-specific synaptic functional changes further, we conducted a detailed analysis of SC neurons, including motor neurons, which exhibited the most significant differences in gene expression. The primary population classified as ‘Neuron’ in the SC was further divided into 13 excitatory (EX_1-EX_13), seven inhibitory (INH_1-INH_7) and one motor neuron (MN_1) subpopulation(s) (Fig. 4C). In an integrated analysis of the human spinal spatial transcriptome,^30^ both excitatory and inhibitory neurons showed subsets located dorsally and ventrally (e.g. EX_1 dorsally, EX_9 ventrally; INH_4 dorsally, INH_7 ventrally), whereas MN_1 was specifically located in the ventral horn of the SC (Fig. 4D and Supplementary Fig. 2A). In MN_1, which exhibited the highest number of DEGs in ALS, *GRM5* and *NAALAD2* were upregulated, whereas *CALB1* was downregulated (Fig. 4E). GO enrichment analysis of these DEGs revealed the activation of pathways related to the regulation of chemical synaptic transmission and glutamate receptor signalling and diminished responses to cadmium and copper ion stress in ALS (Fig. 4F).

The observed gene expression differences were validated using immunohistochemistry on paraffin-embedded lumbar SC sections from patients with ALS, and differences were assessed at the protein level. GRM5 expression was significantly upregulated in the spinal ventral horn neurons of patients with ALS (Fig. 4G and H). Further, to explore the relationship between GRM5 and TDP-43, we conducted western blotting of primary cultured mouse cortical neurons under TDP-43 knockdown conditions. The results showed that TDP-43 downregulation was accompanied by increased GRM5 expression (Fig. 4I and J). These findings indicate that the most pronounced gene expression changes in ALS occurred in spinal motor neurons, with a potential link between TDP-43 dysfunction and GRM5 regulation.

### Microglia-specific chromatin accessibility and transcription factor activity linked to ALS risk SNPs

**Figure 5:**
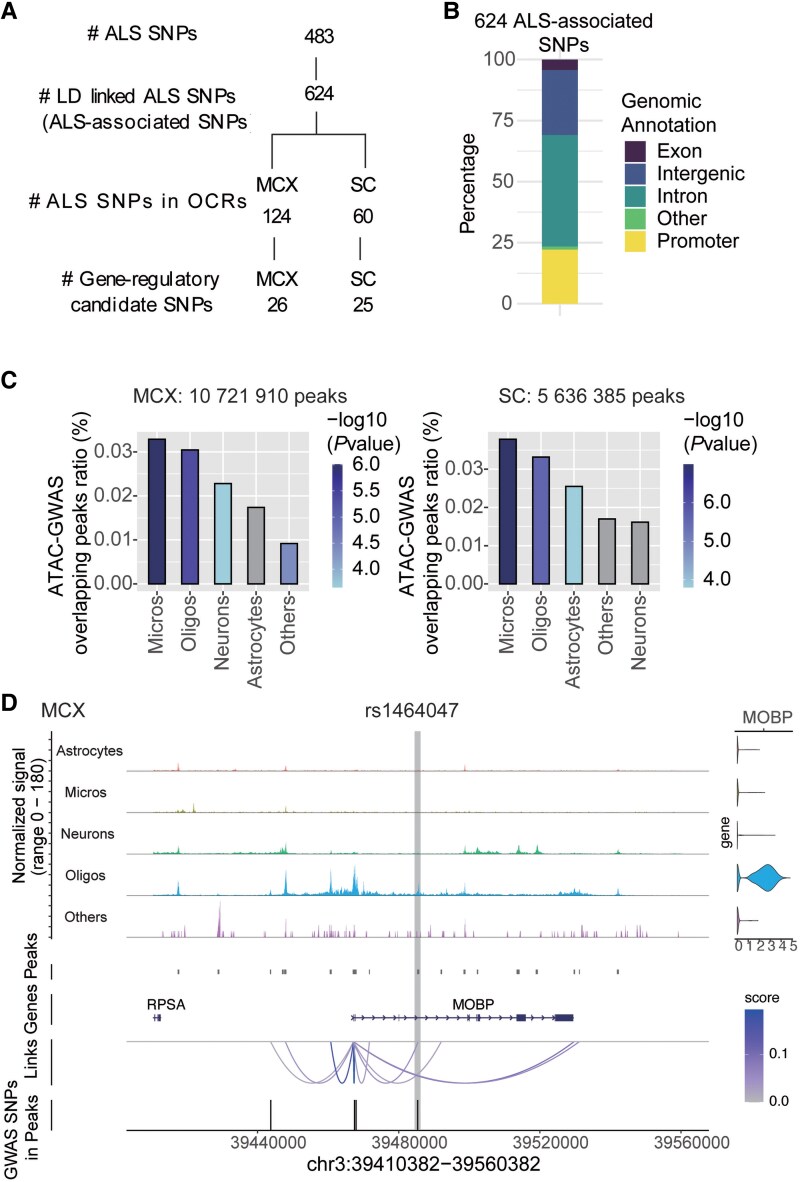
**Multiome–GWAS integrated analysis reveals amyotrophic lateral sclerosis-associated SNPs with microglia-specific activation.** (**A**) Overview of SNP selections. From the amyotrophic lateral sclerosis (ALS) genome-wide association study (GWAS) summary statistics,^8^ 483 SNPs with a *P* < 5 × 10^−8^ were extracted. Additional SNPs in linkage disequilibrium (*R*^2^ > 0.9) were included, resulting in 624 ALS-associated SNPs. Furthermore, SNPs overlapping with peaks detected in both the motor cortex (MCX) and spinal cord (SC) [ALS SNPs in open chromatin regions (OCRs)] were identified. SNPs correlated with nearby gene expression were classified as candidate gene-regulatory SNPs. (**B**) Distribution of gene annotations for ALS-associated SNPs. The *y*-axis shows the percentage of each annotation relative to the total. (**C**) Frequency of ALS SNPs in OCRs at the peaks called for each cell type. The frequency of overlap is shown for each cell type in the MCX (*right*) and SC (*left*). Significance is shown in colours, and cell types with non-significant differences are shown in grey. (**D**) Representative chromatin accessibility sequence tracks with candidate gene-regulatory SNPs around the *MOBP* gene loci. Peaks significantly correlated with the gene expression were indicated by the purple arc (*P* < 0.1, score > 0.01). Significant associated SNPs (*P* < 5 × 10^−8^) within ATAC (assay for transposase-accessible chromatin) peaks are shown at the bottom. SNP = single nucleotide polymorphism.

To investigate ALS risk variants further, we performed an integrative analysis using GWAS and epigenomic data. Quality-controlled cells that were also used for snRNA-seq analysis were selected for snATAC-seq profiling. These included four patients with ALS and four controls, with 35 739 nuclei and 42 clusters from the MCX and 26 061 nuclei and 43 clusters from the SC (Supplementary Fig. 7A and B). First, we created a list of 483 susceptible SNPs from ALS GWAS^8^ (Supplementary Table 3) and conducted an expansion based on linkage disequilibrium (LD), identifying neighbouring SNPs with a high correlation (*R*² > 0.9, using the 1000 Genomes Project Phase 3 data). Thus, we identified 624 ALS-associated SNPs (Fig. 5A and Supplementary Table 4). Of them, 95.67% were located in non-coding regions (Fig. 5B). As a large proportion of ALS-associated SNPs are located in non-coding regions,^8^ integrating chromatin accessibility data with RNA-based approaches may help refine the interpretation of these variants and uncover their potential regulatory functions.

To determine the cell types that activate ALS-associated SNPs, we analysed our snATAC-seq data. Peak calling was performed to identify the OCRs for the five major cell types in both the MCX and SC. They included astrocytes, neurons, oligodendrocytes (including OPCs), microglia (including macrophages) and other cells (e.g. pericytes, endothelial cells, perivascular fibroblasts, lymphocytes and ependymal cells). Overall, we detected 10 721 910 chromatin accessibility peaks in the MCX and 5 636 385 peaks in the SC (Supplementary Fig. 7C and D). Among them, 65.2% in the MCX and 54.2% in the SC were specific to a single-cell type (Supplementary Fig. 7E and F). We then calculated the overlap between cell-type-specific snATAC-seq peaks and ALS-associated SNPs to identify ALS SNPs in OCRs that represented 19.9% of the total ALS-associated SNPs (124 SNPs) in the MCX and 9.6% (60 SNPs) in the SC (Fig. 5A and Supplementary Tables 5 and 6). Furthermore, more than half of these SNPs were detected in the OCRs of only one or two cell types, with 85.5% in the MCX and 76.6% in the SC, suggesting cell-type-specific localization (Supplementary Fig. 8A and B). Additionally, statistical analysis of the cell-specific distribution of ALS SNPs in OCRs revealed that a significant number of peaks containing these SNPs were specifically enriched in microglia-specific peaks (*P* = 9.83 × 10^−7^ in MCX, *P* = 1.00 × 10^−7^ in SC) and oligodendrocyte-specific peaks (*P* = 4.47 × 10^−6^ in MCX, 2.53 × 10^−6^ in SC) in both the MCX and SC. In the MCX, a significant overlap was also observed in neurons and other cell types, whereas in the SC, a significant overlap was found in astrocytes (Fig. 5C).

Next, to identify SNPs potentially involved in transcriptional regulation, we investigated ‘gene-regulatory candidate SNPs’, which showed significant correlations with neighbouring RNA expression within the ALS SNPs in OCRs. Consequently, we detected 26 candidate gene-regulatory SNPs in the MCX and 25 in SC (Supplementary Tables 7 and 8). These SNPs were identified in genes such as *MOBP*, *MOB3B* (adjacent to *C9orf72*) and *TBK1* (Fig. 5D and Supplementary Fig. 8C–E). For example, rs61933209, located in the intron of *TBK1*, showed the highest chromatin accessibility in microglia and was predicted to regulate *RASSF3* expression in both the MCX and SC (Supplementary Fig. 9A and B). Several SNPs, including rs1464047 near the *MOBP* gene, which encodes myelin-associated oligodendrocyte basic protein, overlapped with gene regulatory regions in oligodendrocytes (Fig. 5D and Supplementary Fig. 8C and D). Overall, by integrating multiome data from the MCX and SC with GWAS summary statistics, we catalogued the cell-specific associations of ALS susceptibility.

## Discussion

In this study, we conducted multi-omics profiling using fresh-frozen tissues from post-mortem patients with ALS and controls to gain critical insights into ALS pathology. We investigated the cell-type specificity of ALS susceptibility genes and revealed enrichment in spinal motor neurons, microglia and astrocytes. Furthermore, we identified the dysregulation of oligodendrocyte subpopulations in the SC, which are involved in neuroprotection and synaptic stabilization via *PTN* and *NRXN*. We also found increased CCC centred around motor neurons in the SC. Additionally, significant gene expression changes were observed in spinal motor neurons, supporting the idea that glutamate overactivation may contribute to ALS pathology.

Numerous studies have suggested that oligodendrocytes are involved in ALS pathogenesis. The oligodendrocyte subpopulation identified in this study is involved in neuroprotection and synaptic stabilization, suggesting that specific mature oligodendrocytes may be decreased in ALS. Oligodendrocytes have a close relationship with motor neuron axons, and in ALS, some OPCs fail to fully differentiate into mature oligodendrocytes, leading to dysregulation of metabolic support and axonal myelination.^50-53^ Moreover, whereas microglial and astrocytic activation have been widely reported, oligodendrocytes have been shown to exhibit reduced activity or dysfunction.^49,54^ Our findings suggest that this impaired differentiation may contribute to a reduction in oligodendrocytes associated with synaptic stabilization in ALS pathology.

Although non-cell-autonomous deterioration contributes to ALS pathogenesis, some changes in non-neuronal cells may result from cell–cell communication with neurons.^3^ In this study, we present data suggesting differential changes in cell–cell interactions (CCI) between neurons and non-neurons in both the MCX and SC in patients with ALS. In the MCX, the overall CCI decreased, whereas in the SC, interactions were centred on motor neurons, and those with astrocytes, OPCs and oligodendrocytes were particularly upregulated. Specifically, glutamate and *NRXN* signalling via *NLGN1* were upregulated. Although neuronal excitotoxicity caused by excess glutamate is well known in ALS pathology,^45-47^ our findings also suggest the involvement of excessive glutamate signalling in the SC in ALS. Further, the *NRXN*-*NLGN1* pathway is involved in regulating the function of glutamatergic *N*-methyl-D-aspartate (NMDA) receptors,^55,56^ which may be linked to alterations in glutamate signalling. Furthermore, our data indicate that these network changes are directed toward motor neurons and involve communication from neurons to glial cells, highlighting the necessity of considering disruptions in the neuron-glia crosstalk in ALS.

Moreover, a comparison of gene expression changes across cell types between ALS and control samples revealed prominent differential gene expression in the SC, with significant upregulation of glutamate-related genes in spinal motor neurons. In particular, *GRM5*, which encodes the mGluR5 protein and belongs to group 1 metabotropic glutamate receptors along with mGluR1, was upregulated in the spinal motor neurons of patients with ALS. Furthermore, experiments using primary cultured cells demonstrated that *GRM5* overexpression was linked to TDP-43 dysfunction. Glutamate excitotoxicity is a key pathological factor in ALS.^57^ Group I metabotropic glutamate receptors (mGluR1 and mGluR5) may play a key role in ALS pathology,^58-62^ although the detailed mechanisms remain unclear. Our results support these findings and highlight *GRM5* as a potential therapeutic target.

Our GWAS-integrated analysis revealed that ALS-associated genetic risk is not limited to motor neurons but also accumulates in glial cells. We observed a notable accumulation of genetic susceptibility in the microglia of both the MCX and SC, along with that in motor neurons. When evaluating chromatin accessibility across different cell types, microglia showed a concentration of ALS-related genetic susceptibility loci. Microglial activation in the ALS brain has been consistently reported, and such changes may be underpinned by genetic factors.^49,54^ The inflation of ALS risk loci enrichment in microglia may be partially explained by the tendency of immune cell-associated disease risk variants to fall within enhancer regions.^63,64^ Altogether, these findings suggest that microglia may contribute to ALS pathogenesis through susceptibility conferred by common variants.

Our analysis also highlights several other key genes with potential roles in ALS pathology. *ERBB4*, previously reported as an ALS-associated gene, plays essential physiological roles in neurons, astrocytes and oligodendrocytes, contributing to critical functions in these cells.^65-67^ These findings suggest that *ERBB4* plays a multifaceted role in ALS pathogenesis. Additionally, we identified overlaps between cell-specific gene regulatory regions and GWAS SNPs at loci such as *MOBP*, *MOB3B* and *RASSF3*. While these findings remain suggestive due to the lack of experimental validation in this study, they highlight the contribution of gene regulation to sALS susceptibility and underscore the value of our resource for investigating such mechanisms.

In conclusion, this study demonstrates the potential involvement of glutamate overactivation in spinal motor neurons in ALS pathology and highlights the genetic susceptibility of microglia. Our findings provide valuable insights into the mechanisms underlying ALS, particularly the roles of motor neuron–glial interactions, which may inform future strategies for precision medicine.

In this study, sn-multiome technology allowed us to capture changes in gene expression and chromatin accessibility in a cell-type-specific manner. However, this study has a few limitations that must be acknowledged. First, the number of samples was small, making it difficult to identify rare cell populations and potentially limiting the statistical power to detect subtle but biologically meaningful differences in cell frequencies and gene expression. Additionally, the yield of certain cell types, including motor neurons, was low, which limited our ability to capture chromatin accessibility changes at the cell-type level in ALS. Future studies with larger sample sizes may allow for a more comprehensive exploration of specific cell types and cell-type-specific gene changes. Moreover, although our analysis identified chromatin accessibility patterns associated with ALS-related SNPs and cell-type-specific genetic susceptibilities from omics data, functional validation and clarification of their biological significance are needed in future studies.

## Supplementary Material

awaf426_Supplementary_Data

## Data Availability

Raw sequence data have been deposited at the DNA Data Bank of Japan (DDBJ) under the Accession ID JGAS000852. The codes are available on GitHub (https://github.com/ertakeuchi/ALS_project). The processed snRNA/ATAC-seq data and full DEG statistics are available in figshare (DOI: 10.6084/m9.figshare.29628653) and CZ CELLxGENE (https://cellxgene.cziscience.com/collections/0986e4cd-7a58-405d-9b91-4b199bb4124e).
