# Supplementary material for: Single-nucleus multiome shows motor neuron glutamate overactivation in amyotrophic lateral sclerosis
Source: Brain. 2025 Nov 11;149(7):2480–94. doi: 10.1093/brain/awaf426 (PMC13337230; doi:10.1093/brain/awaf426)
Supplement: awaf426_Supplementary_Data [file awaf426_supplementary_data.zip › brain-2025-00589-File008.pdf]

Supplementary Methods

Genomic DNA extraction and whole genome sequencing

Genomic DNA (gDNA) was extracted from frozen brain tissue of patients with ALS and control individuals using the Qiagen QIAamp DNA Mini Kit, according to the manufacturer's protocol. Library preparation was performed using the TruSeq DNA PCR-Free Kit (Macrogen, Japan), and whole-genome sequencing was conducted on the NovaSeq6000 platform with 150-bp paired-end reads. FASTQ files were checked for quality using FastQC, and low-quality reads were removed using Trimmomatic. Quality-checked reads were aligned with GRCh38 using the Burrows–Wheeler Aligner (http://bio-bwa.sourceforge.net/). Variants were identified using the Genome Analysis Toolkit (GATK v.4.1.0) HaplotypeCaller and annotated using ANNOVAR. The clinical significance of the variants was determined based on the American College of Medical Genetics and Genomics (ACMG) guidelines[1] and further refined by manual adjustments based on a thorough review of relevant literature. Patients with ALS were confirmed to have no family history of ALS, and genotyping confirmed the absence of ALS-associated risk factors (such as mutations in *SOD1*, *TARDBP*, *FUS*, *NEK1*, *GRN*, *TBK1*, *and ERBB4*); for *C9orf72*, repeat primed PCR was used to confirm the genotype.

Nuclei isolation

For both ALS and control samples, fresh frozen MCX or half a segment of the L4/L5 region of the lumbar SC was used for nuclei isolation. Isolation was performed using a density gradient centrifugation method based on 10X Genomics nuclear isolation (CG000375 Rev C) and Optiprep™ protocols. Briefly, 60–180 mg of the MCX or half a segment of the lumbar SC was placed in a Dounce homogenizer containing 500 μL of lysis buffer (10 mM Tris-HCl, 10 mM NaCl, 3 mM $MgCl_2$, 0.1% Tween-20, 0.1% NP40, 0.01% Digitonin, 1% BSA, 1mM DTT, and 0.2 U/μL RNase inhibitor). After homogenizing with 15 strokes of a pestle, the lysate was diluted in 1 mL of wash buffer (10 mM Tris-HCl, 10 mM NaCl, 3 mM $MgCl_2$, 1% BSA, 0.1% Tween-20, 1mM DTT, and 0.2 U/μL RNase inhibitor) and passed through a 100-μm strainer. The filtered lysate was centrifuged at 500 ×*g* for 5 min at 4 °C. After centrifugation, the pellet was resuspended in 350 μL sucrose buffer A (250 mM sucrose, 25 mM KCl, 5 mM $MgCl_2$, and 20 mM Tricine-KOH [pH 7.8]) and 450 μL sucrose buffer B (150 mM KCl, 30 mM $MgCl_2$,

and 120 mM Tricine-KOH [pH 7.8]). Then, 700 µL of 35% iodixanol buffer (60% Optiprep, 150 mM KCl, 30 mM $MgCl_2$, 120 mM Tricine-KOH [pH 7.8], and 0.2 U/µL RNase inhibitor) was layered on the bottom of a centrifuge tube, 700 µL of 29% iodixanol buffer was layered above the 35% layer, and the sample was layered above the 29% layer. The tube was centrifuged at 7000 rpm for 20 min at 4 °C. The nuclei at the bottom of the middle layer were extracted, resuspended in 3 mL of wash buffer, and centrifuged at 500 ×*g* for 5 min at 4 °C. After centrifugation, the pellet was resuspended in 1 mL of wash buffer and centrifuged at 500 ×*g* at 4°C. The washing step was repeated twice. After centrifugation, the pellet was resuspended in wash buffer and passed through a 40-µm strainer. The nuclei were immediately used for droplet-based single-nucleus RNA sequencing and ATAC sequencing.

Droplet-based single-cell sequencing

Droplet-based snRNA sequencing libraries were constructed using the Chromium Next GEM Single Cell 3′ reagent kit v3.1 (10× Genomics) or Chromium Next GEM Single Cell Multiome ATAC + Gene Expression reagent according to the manufacturer's protocol. Libraries were sequenced on a NovaSeq6000 platform (Illumina). Raw sequenced reads were processed using Cell Ranger software (v6.0.0). Cell ranger counts with the include-introns option were used for RNA libraries, and Cell ranger ARC count was used for multiome libraries with the pre-built reference refdata-gex-GRCh38-2020-A, which was downloaded from the 10× Genomics website.

Pipeline for snRNA-seq analysis

The R package Seurat (v4.3.0) [2] was used to process the count data. Low-quality cells were excluded based on the criteria of nFeature (<200 or >10,000) and mitochondrial gene contamination exceeding 5%. Additionally, demuxlet[3] was used for sample demultiplexing using germline mutations, only extracting variants with an allele frequency (AF) between 0.1 and 0.9. Only cells with a posterior probability greater than 99% were retained and labeled according to their patient origin. DoubletFinder[4] (v2.0.3) was used to exclude cells predicted as doublets. The expected doublet rate necessary for DoubletFinder was manually set based on the number of cells obtained from each sequencing run, using the cost per cell as a reference (https://satijalab.org/costpercell/). Data transformation was conducted using the *SCTransform* function for scaling, and highly variable genes (HVGs) were identified in the brain (3000 genes) and SC (3000 genes) using the *SelectIntegrationFeature* function. Principal component

analysis (PCA) was performed on gene expression data, followed by batch correction using Harmony (v0.1.1).[5] Dimensionality reduction was achieved using uniform manifold approximation and projection (UMAP) based on the first 50 harmony-corrected principal components from the brain and SC. Neighbor graphs were computed using the *FindNeighbors* function, and clustering was performed with the *FindClusters* function using the Leiden algorithm.

For cell population identification, DEGs for each cluster were calculated using the *FindAllMarkers* function and compared with known cell type-specific markers for annotation. Initial clustering identified five major cell types in the brain and five in the SC. For more detailed clustering of major cell types, the following subsets were analyzed separately: astrocytes, neurons, oligos (oligodendrocytes, OPCs), micros (microglia, macrophages), and others (pericytes, endothelial cells, perivascular fibroblasts) in the brain; and astrocytes, neurons, oligos (oligodendrocytes, oligo precursor cells), micros (microglia, macrophages), and others (lymphocytes, pericytes, endothelial cells, ependymal cells) in the SC. For each subset, SCT scaling was reapplied to the RNA count data, and HVGs were identified (2000 genes in the brain and 2000 genes in the SC, followed by PCA, Harmony batch correction, neighbor graph computation, UMAP dimensionality reduction, and Leiden clustering. Clusters in which multiple cell type-specific marker genes were highly expressed were identified as potential doublets and subsequently removed. This process was iterated by adjusting the HVGs between 1000 and 5000, resulting in the identification of 42 brain and 43 SC subsets. For the final annotation, the brain subsets were referenced against the Azimuth[2]–brain cortex annotation, and the SC subsets were annotated based on previously published data.[6] To identify statistically credible changes in cell clusters, cell proportions were compared using scCODA[7]. scCODA applies Bayesian inference to assess changes in cell-type composition, providing credibility scores and highest density intervals (HDIs) as outputs. The inference was performed with an FDR threshold of 0.1. To investigate the biological characteristics of each cell subset, enrichment analysis was performed using enrichGO (ont = "BP") from ClusterProfiler[8] (v4.2.2), with the DEGs for each cell type obtained using FindAllMarkers as the input.

Gene-level downstream analysis for the snRNA-seq dataset

We conducted a differential gene expression analysis between patients with ALS (n = 6) and controls (n = 6). Pseudo-bulk analysis was performed by aggregating the raw gene count data by cell type in each sample. Genes expressed in at least ten cells were included in the analysis.

Differential gene expression testing was performed using DESeq2,[9] which applies a generalized linear model (GLM) based on a negative binomial distribution. Age and sex were included as covariates in the model, and significant genes were identified based on adjusted p-values (padj < 0.2) using the Wald test. To explore the biological functions of DEGs, we used enrichGO (ont= "BP") from ClusterProfiler[8] (v4.2.2) for biological pathway enrichment scoring and visualized the results using the *compareCluster* function. We also compared the expression of 59 manually curated ALS-related genes[10–12] across different cell types. To observe cell type-specific expression patterns, we performed PCA on the matrix of cell types by averaging gene expression using the *prcomp* function in the stats package. The results were visualized using the *autoplot* function of the ggfortify package.[13] Genes were classified into three groups based on their PC1 and PC2 values using k-means clustering (k = 3).

Cell–cell interaction analysis

We used NeuronChat[14] to evaluate changes in CCC between the ALS and control groups. NeuronChat estimates the abundance of ligands and targets based on the expression levels of all genes associated with 190 ligand–target interaction pairs in a neuron-specific database (https://github.com/Wei-BioMath/NeuronChatAnalysis2022/tree/main/NeuronChatDB_table/interactionDB_human.txt). As input for NeuronChat, we used the values obtained by normalizing RNA counts with the *NormalizeData* function, which divides RNA counts by the total counts for each cell, multiplies them by a scaling factor (the default is 10,000), adds a pseudo-count of 1, and then applies a natural logarithmic transformation. The CCC strength between the two cell types was defined as the product of ligand abundance in one cell group and target abundance in another cell group. For non-peptide neurotransmitters, the expression levels of synthetic enzymes and transporters were also incorporated in the computation of communication strength, as described in the original NeuronChat publication. Significant CCCs within the ALS and control groups were determined using permutation tests as implemented in NeuronChat (20 permutations), followed by multiple test correction, and considered links with an adjusted P-value (FDR < 0.05) to be significant. Receptors from NeuronChat were manually classified into biological families. For both SC and MCX, gene sets corresponding to each family were used to calculate module scores per cell using the AddModuleScore function in Seurat. Differences in module scores between ALS and HC were evaluated within each cluster and family using Welch's t-test, followed by FDR correction. Results were visualized as a dot plot.

GWAS integration analysis with the snRNA-seq dataset

We used the single-cell disease relevance score (scDRS)[15] to integrate the gene expression data obtained from snRNA-seq with polygenic disease information from GWAS to identify the cell populations associated with ALS. Specifically, for gene and SNP locations, we downloaded the reference gene locations (build 37) and Phase 3 of the 1,000 Genomes European dataset from the MAGMA website (https://cncr.nl/research/magma/). Additionally, we obtained the ALS GWAS summary statistics GCST90027164[16] downloaded from the NHGRI-EBI GWAS Catalog, which describe the meta-analysis results for ALS. The cohort included 27,205 European ancestry cases and 110,881 European ancestry controls. Summary statistics were used to weight genes using MAGMA (v1.10),[17] and the top 1000 disease-predicted genes were extracted. Before running MAGMA, we removed SNPs within the HLA region (25–34 Mb on chromosome 6) because of its complex linkage disequilibrium structure. Single SNPs that were absent from the HapMap3 dataset were excluded. For each of these 1000 genes, we used scDRS to score the total expression level in each cell and compared these scores with 1000 matched control sets to detect cell types with significant heritability enrichment. As controls, we used the selected four gs files (with GWAS MAGMA z-score weights), multiple sclerosis (MS), schizophrenia (SCZ), and height, from (https://figshare.com/articles/dataset/scDRS_data_release_030122/19312583?file=34300898).

Spatial transcriptome data analysis

Spatial transcriptome data of the human SC were downloaded from https://als-st.nygenome.org/.[18] The pre-annotated regions were aligned across slides by selecting a reference slide and optimizing an affine transformation to align all other slides with this reference. First, a single reference section was selected. For each region, the nearest-neighbor distance for each data point was calculated using *KDTree* in SciPy, and the affine transformation was optimized to minimize the total distance. Optimization was performed using the *optimize.minimize*(method= "SLSQP") function in SciPy. Cell deconvolution of Visium samples using Cell2location[19] was performed according to tutorial guidelines (https://cell2location.readthedocs.io/en/latest/notebooks/cell2location_tutorial.html). The scRNA-seq reference was filtered (*cell2location.util.filtering.filter_genes* with the options cell_count_cutoff=5, cell_percentage_cutoff2=0.03, nonz_mean_cutoff=1.12) and prepared

(*cell2location.models.RegressionModel.setup_anndata* with the options batch_key='sampleID,' labels_key='celltype_3rd'). A regression model was created using cell2location.models.RegressionModel and trained (model training with max_epochs=250). The cell proportions were inferred for each Visium sample at each time point. In the inference step, a model for the Visium sample was created using *cell2location.models.Cell2location* (N_cells_per_location=30, detection_alpha=20) and trained (max_epochs=30000). The results were exported and used as cell proportions.

Mutiome data and GWAS integration analysis

For preprocessing, snATAC-seq data were analyzed using Signac[20] after assigning snATAC-seq annotations based on shared barcodes obtained from RNA profiling. Peaks located outside standard chromosomes and within blacklist regions were excluded using the *keepStandardChromosomes* and *subsetByOverlaps* functions. The nucleosome signal for each cell was calculated using the *NucleosomeSignal* function in Signac, followed by the calculation of enrichment scores using *TSSEnrichment*. Cells with a TSS score > 1 and nucleosome signal < 2 were retained for downstream analysis. Peak calling was performed using MACS2.[21] A genomic region × cell count matrix was created using the FeatureMatrix function. After merging all project data, variable features were identified using *FindTopFeatures*(min.cutoff = 10), followed by normalization with *RunTFIDF* and dimensionality reduction using *RunSVD*. Batch correction and data integration were performed using Harmony[5] (v0.1.1). To detect cell type-specific marker peaks, the FindAllMarkers function was used with the option logfc.threshold = 1, and the top 10 peaks were visualized using the DoHeatmap function. We performed integrative GWAS analysis using GCST900271648.[16] SNPs with a p-value of less than 5e-08 were extracted, yielding 483 SNPs. Next, LD expansion was performed using PLINK software. SNPs with strong LD ($R^2 > 0.9$) within 10 kb of significant SNPs were included with reference to the European population of the 1000 Genomes Project. Subsequently, the UCSC liftover was used to convert the coordinates from GRCh37 to GRCh38. The reference chain file was downloaded from https://hgdownload.soe.ucsc.edu/goldenPath/hg19/liftOver/hg19ToHg38.over.chain.gz, excluding alternative assemblies. Only SNPs located on chr1–22, chrX, and chrY were included in the analysis. At this stage, we identified 624 ALS-associated SNPs. To annotate these ALS-associated SNPs with gene information, we used the annotatePeak function from ChIPseeker[22,23] with the TxDb.Hsapiens.UCSC.hg38.knownGene database and a TSS region

of −2000 to +2000 bp. Peak calling was performed using MACS2 for each major cell type to obtain cell type-specific peaks. In the MCX, peaks were called for the following cell types: astrocytes, neurons, oligos (oligodendrocytes, OPCs), micros (microglia, macrophages), and others (pericytes, endothelial cells, perivascular fibroblasts). Similarly, for the SC, peak calling was performed for astrocytes, neurons, oligos (oligodendrocytes, oligo precursor cells), micros (microglia, macrophages), and others (lymphocytes, pericytes, endothelial cells, ependymal cells). To examine the overlap between ALS-associated SNPs and the peaks for each cell type (ALS SNPs in OCRs), we used the findOverlaps function in IRange packages.[24] This allowed us to calculate the proportion of ALS SNPs in the OCRs relative to the peaks in each cell type. For statistical analysis of the overlap between peaks in each cell type and ALS-associated SNPs, we used *bedtools fisher.*[25]

Next, to narrow down the SNPs that were more likely to be involved in gene regulation ("Gene-regulatory candidate SNPs"), we calculated the correlation between each peak and nearby gene expression using the *LinkPeaks* function with distance = 1e6, pvalue_cutoff = 0.1, and score_cutoff = 0.01. Genes within ±100 kb of the peak were included in the correlation analysis. Only peaks with a z-score ≥ 2 were extracted, and the overlap with ALS SNPs in OCRs was examined using *findOverlaps*, allowing us to identify candidate gene-regulatory SNPs. A locus plot was plotted using *locusplotr* [26].

References

1. Richards S, Aziz N, Bale S, et al. Standards and guidelines for the interpretation of sequence variants: a joint consensus recommendation of the American College of Medical Genetics and Genomics and the Association for Molecular Pathology. *Genet Med.* 2015;17(5):405-424.

2. Hao Y, Hao S, Andersen-Nissen E, et al. Integrated analysis of multimodal single-cell data. *Cell*. 2021;184(13):3573-3587.e29.

3. Kang HM, Subramaniam M, Targ S, et al. Multiplexed droplet single-cell RNA-sequencing using natural genetic variation. *Nat Biotechnol*. 2018;36(1):89-94.

4. McGinnis CS, Murrow LM, Gartner ZJ. DoubletFinder: Doublet Detection in Single-Cell RNA Sequencing Data Using Artificial Nearest Neighbors. *Cell Systems*. 2019;8(4):329-337.e4.

5. Korsunsky I, Millard N, Fan J, et al. Fast, sensitive and accurate integration of single-cell data with Harmony. *Nat Methods*. 2019;16(12):1289-1296.

6. Yadav A, Matson KJE, Li L, et al. A cellular taxonomy of the adult human spinal cord. *Neuron*. 2023;111(3):328-344.e7.

7. Büttner M, Ostner J, Müller CL, Theis FJ, Schubert B. scCODA is a Bayesian model for compositional single-cell data analysis. *Nat Commun*. 2021;12(1):6876.

8. Yu G, Wang LG, Han Y, He QY. clusterProfiler: an R package for comparing biological themes among gene clusters. *OMICS*. 2012;16(5):284-287.

9. Love MI, Huber W, Anders S. Moderated estimation of fold change and dispersion for RNA-seq data with DESeq2. *Genome Biol*. 2014;15(12):550.

10. Mejzini R, Flynn LL, Pitout IL, Fletcher S, Wilton SD, Akkari PA. ALS Genetics, Mechanisms, and Therapeutics: Where Are We Now? *Front Neurosci*. 2019;13:1310.

11. Smukowski SN, Maioli H, Latimer CS, Bird TD, Jayadev S, Valdmanis PN. Progress in Amyotrophic Lateral Sclerosis Gene Discovery: Reflecting on Classic Approaches and Leveraging Emerging Technologies. *Neurol Genet*. 2022;8(3):e669.

12. Suzuki N, Nishiyama A, Warita H, Aoki M. Genetics of amyotrophic lateral sclerosis: seeking therapeutic targets in the era of gene therapy. *J Hum Genet*. Published online June 13, 2022. doi:10.1038/s10038-022-01055-8

13. Tang Y, Horikoshi M, Li W. Ggfortify: Unified interface to visualize statistical results of popular R packages. *R J*. 2016;8(2):474.

14. Zhao W, Johnston KG, Ren H, Xu X, Nie Q. Inferring neuron-neuron communications from single-cell transcriptomics through NeuronChat. *Nat Commun*. 2023;14(1):1128.

15. Zhang MJ, Hou K, Dey KK, et al. Polygenic enrichment distinguishes disease associations of individual cells in single-cell RNA-seq data. *Nat Genet*. 2022;54(10):1572-1580.

16. van Rheenen W, van der Spek RAA, Bakker MK, et al. Common and rare variant association analyses in amyotrophic lateral sclerosis identify 15 risk loci with distinct genetic architectures and neuron-specific biology. *Nat Genet*. 2021;53(12):1636-1648.

17. de Leeuw CA, Mooij JM, Heskes T, Posthuma D. MAGMA: generalized gene-set analysis of GWAS data. *PLoS Comput Biol*. 2015;11(4):e1004219.

18. Maniatis S, Äijö T, Vickovic S, et al. Spatiotemporal Dynamics of Molecular Pathology in Amyotrophic Lateral Sclerosis. *Science*. 2019;93(April):89-93.

19. Kleshchevnikov V, Shmatko A, Dann E, et al. Cell2location maps fine-grained cell types in spatial transcriptomics. *Nat Biotechnol*. 2022;40(5):661-671.

20. Stuart T, Srivastava A, Madad S, Lareau CA, Satija R. Single-cell chromatin state analysis with Signac. *Nat Methods*. 2021;18(11):1333-1341.

21. Zhang Y, Liu T, Meyer CA, et al. Model-based analysis of ChIP-Seq (MACS). *Genome Biol*. 2008;9(9):R137.

22. Yu G, Wang LG, He QY. ChIPseeker: an R/Bioconductor package for ChIP peak annotation, comparison and visualization. *Bioinformatics*. 2015;31(14):2382-2383.

23. Wang Q, Li M, Wu T, et al. Exploring Epigenomic Datasets by ChIPseeker. *Curr Protoc*. 2022;2(10):e585.

24. Lawrence M, Huber W, Pagès H, et al. Software for computing and annotating genomic ranges. *PLoS Comput Biol*. 2013;9(8):e1003118.

25. Quinlan AR, Hall IM. BEDTools: a flexible suite of utilities for comparing genomic features. *BIOINFORMATICS APPLICATIONS NOTE*. 2010;26(6):841-842.

26. Levin M. locusplotr: Create Regional Association Plots. R package version 0.5.0, https://github.com/mglev1n/locusplotr. 2024. https://mglev1n.github.io/locusplotr
