# Supplementary material for: Single-nucleus multiome shows motor neuron glutamate overactivation in amyotrophic lateral sclerosis
Source: Brain. 2025 Nov 11;149(7):2480–94. doi: 10.1093/brain/awaf426 (PMC13337230; doi:10.1093/brain/awaf426)
Supplement: awaf426_Supplementary_Data [file awaf426_supplementary_data.zip › brain-2025-00589-File009.pdf]

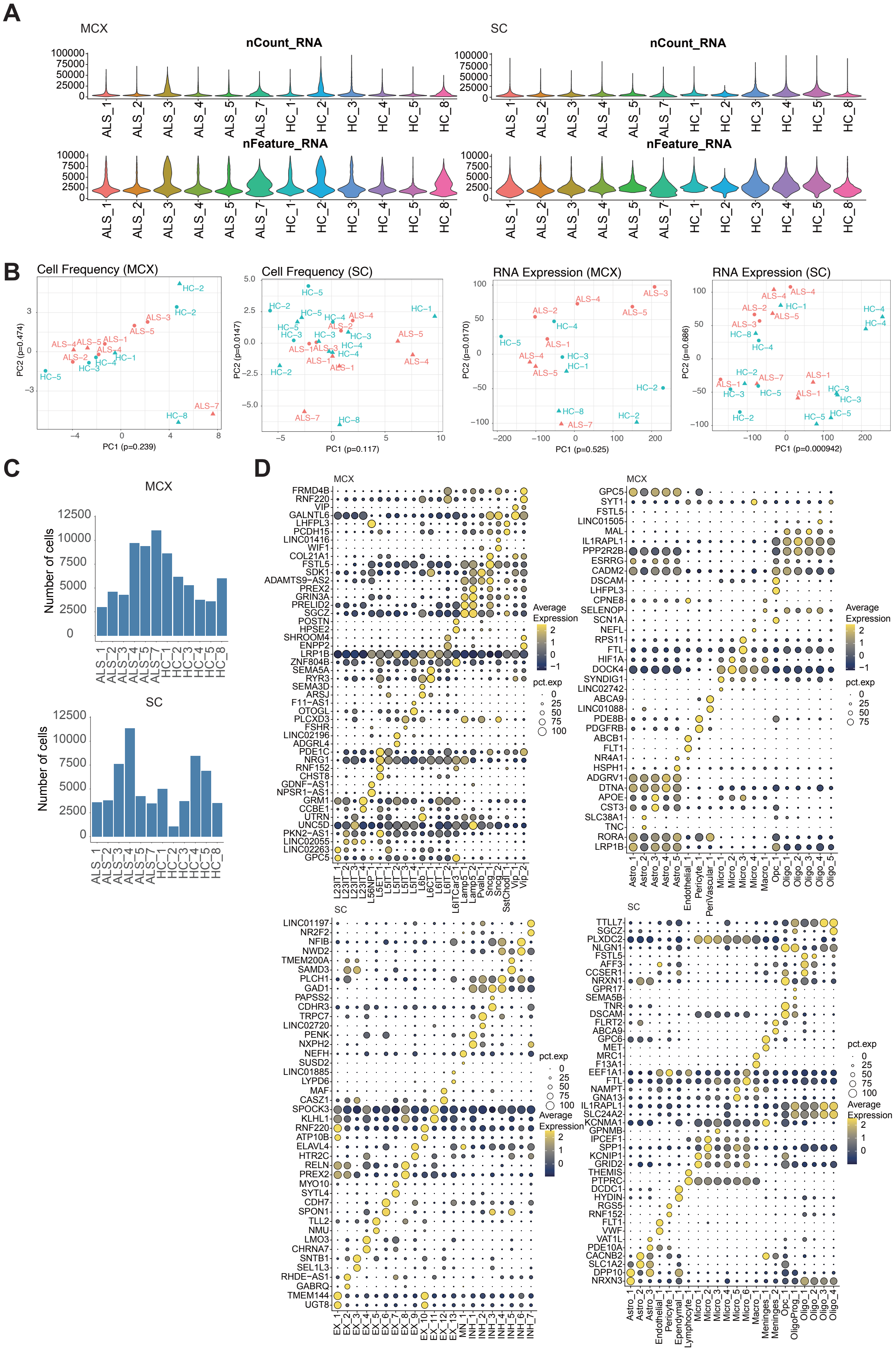

Supplementary Figure 1

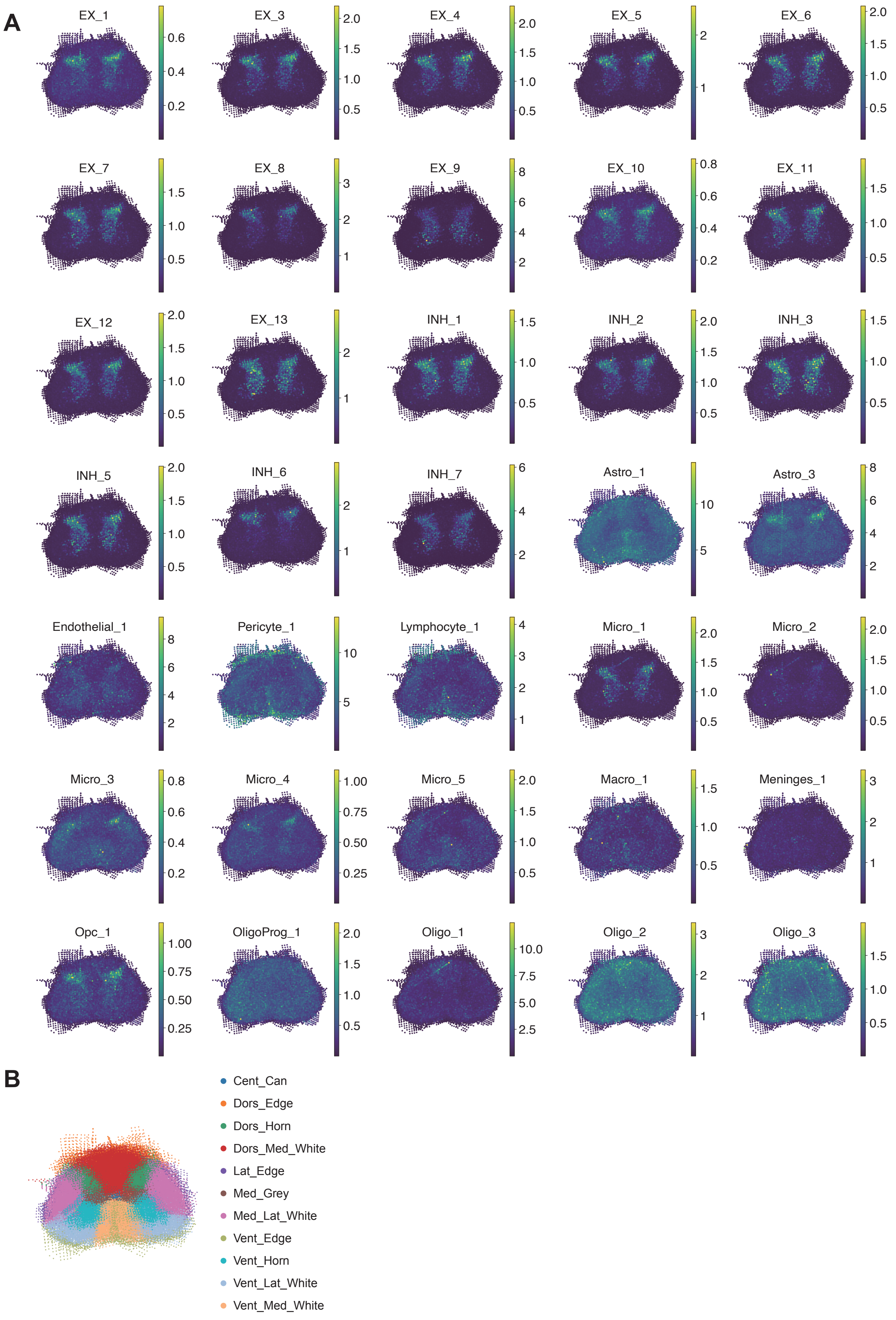

Supplementary Figure 2

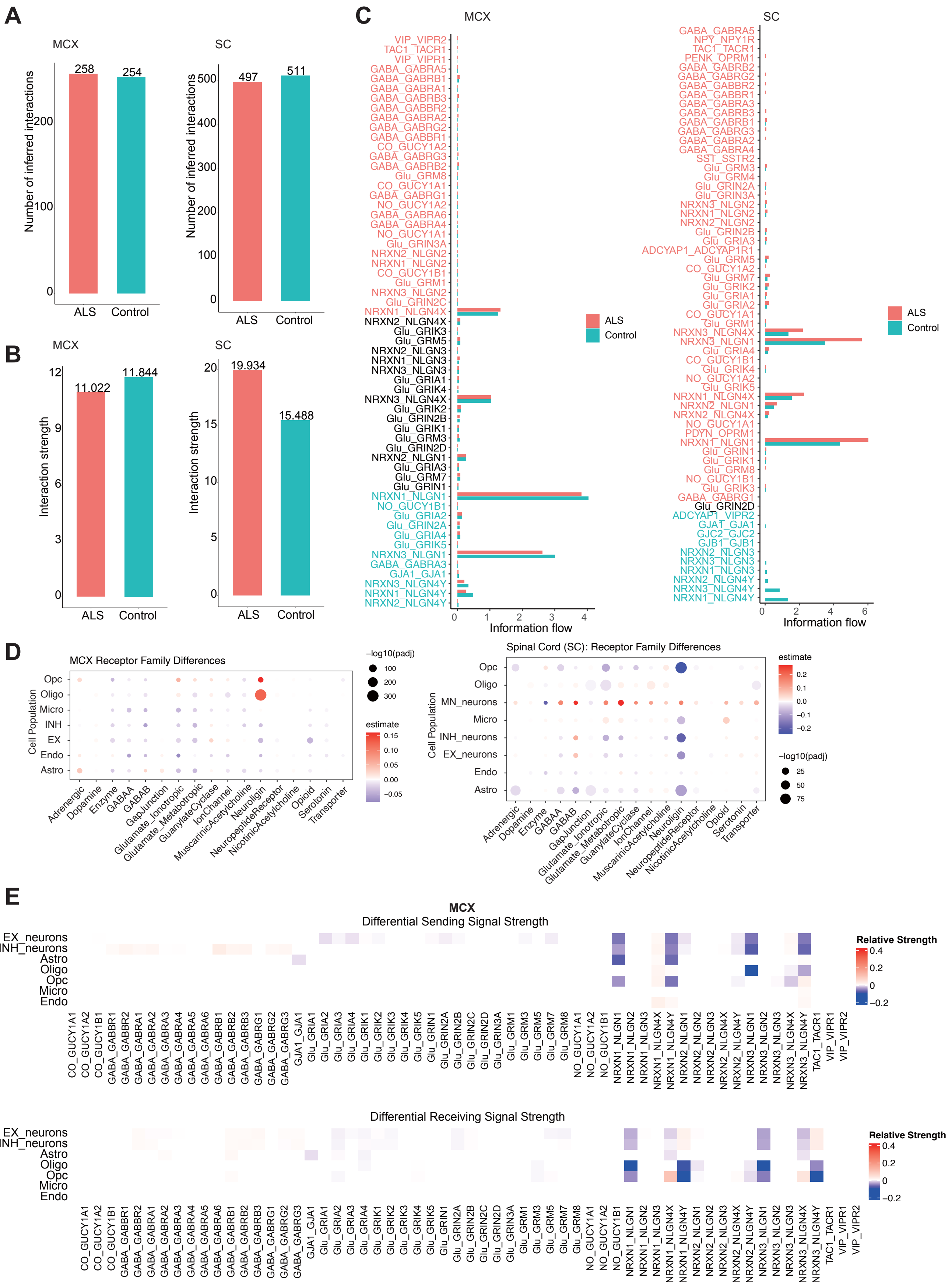

Supplementary Figure 3

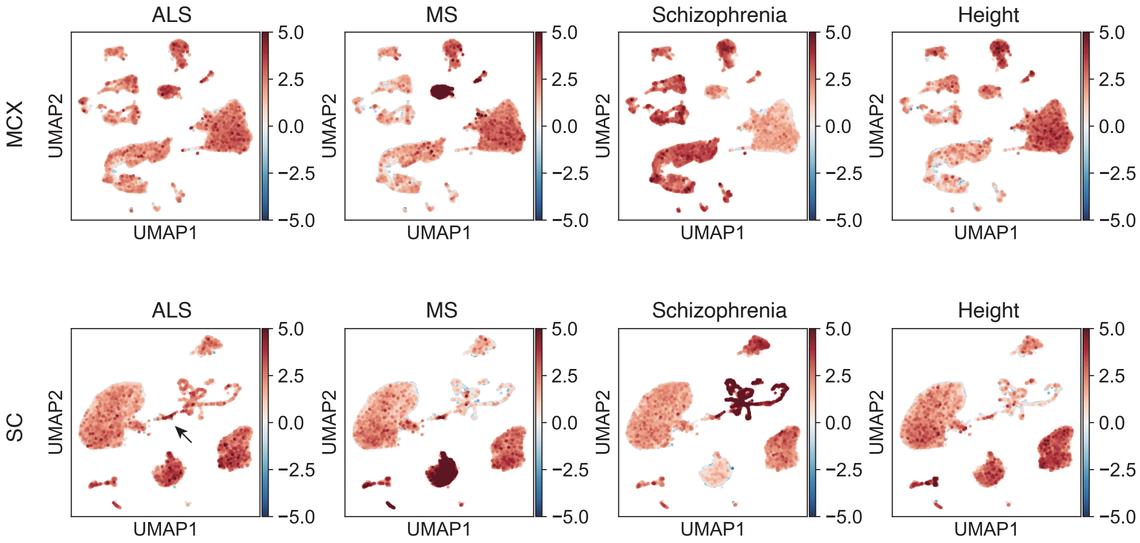

Supplementary Figure 4

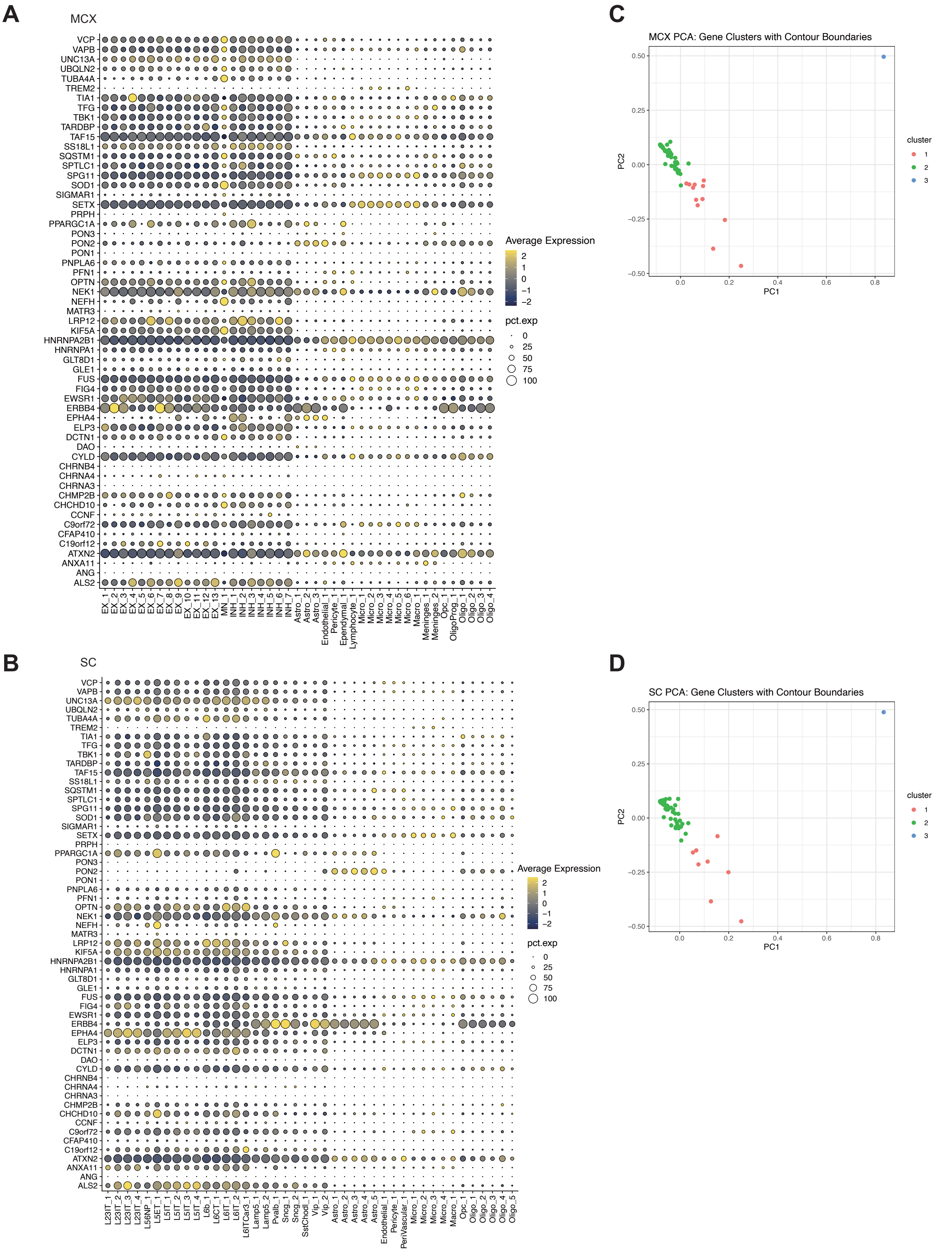

Supplementary Figure 5

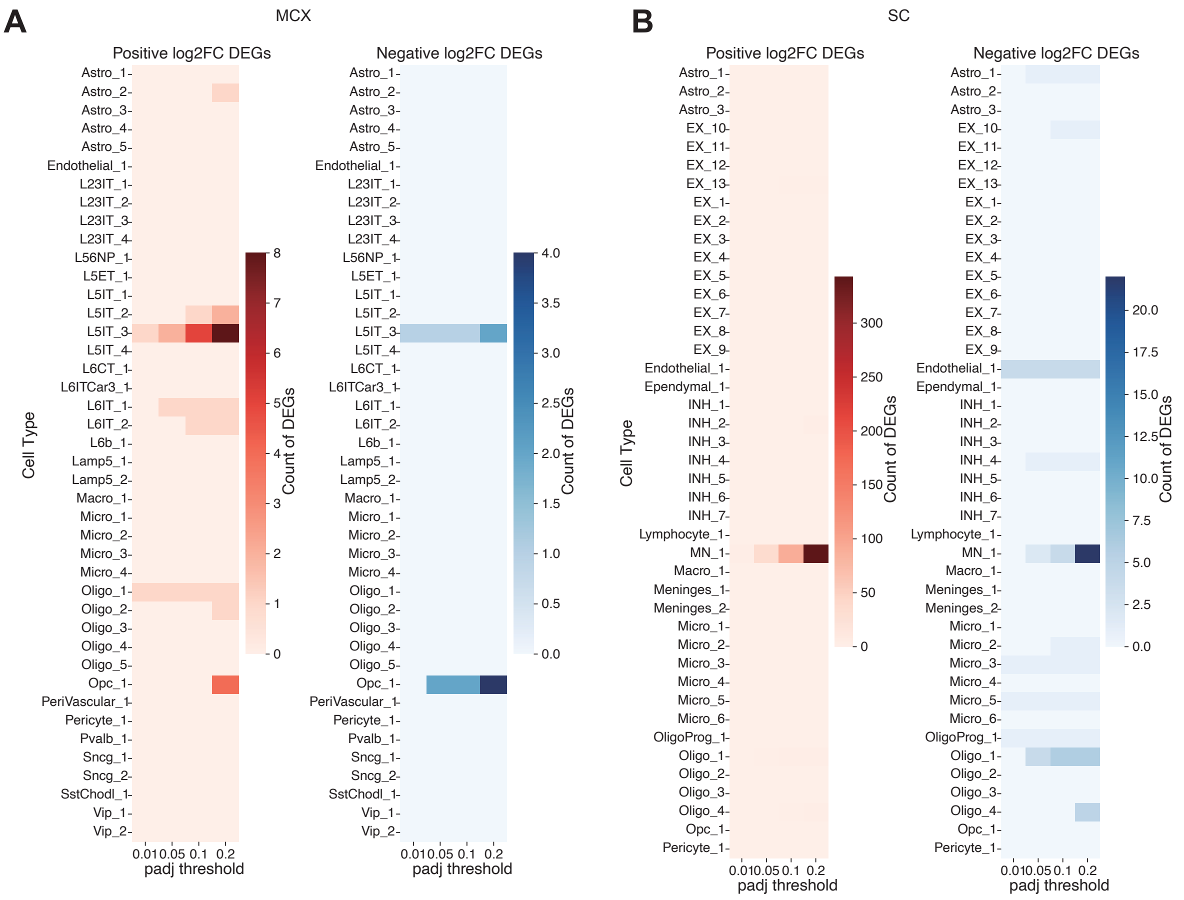

Supplementary Figure 6

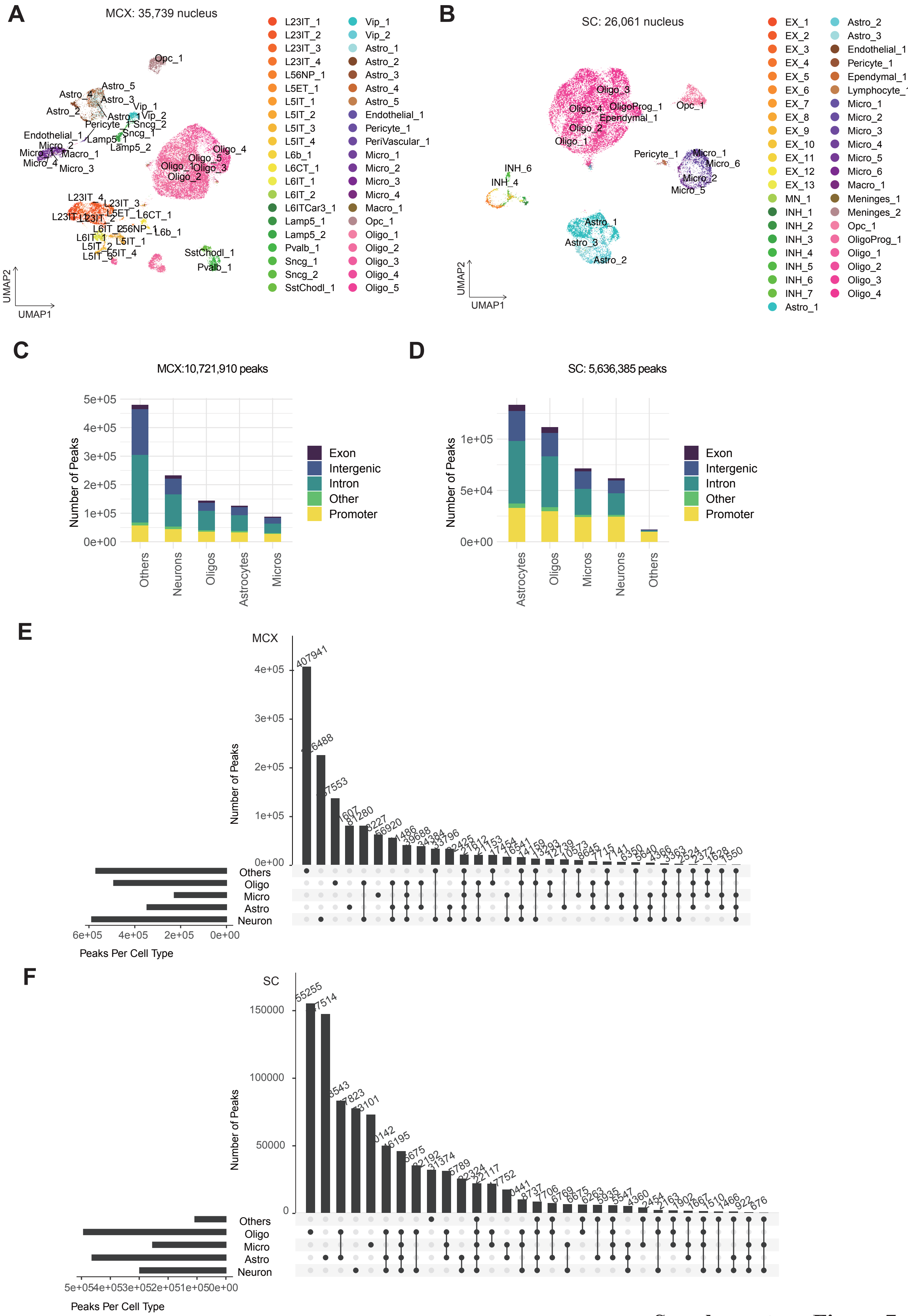

Supplementary Figure 7

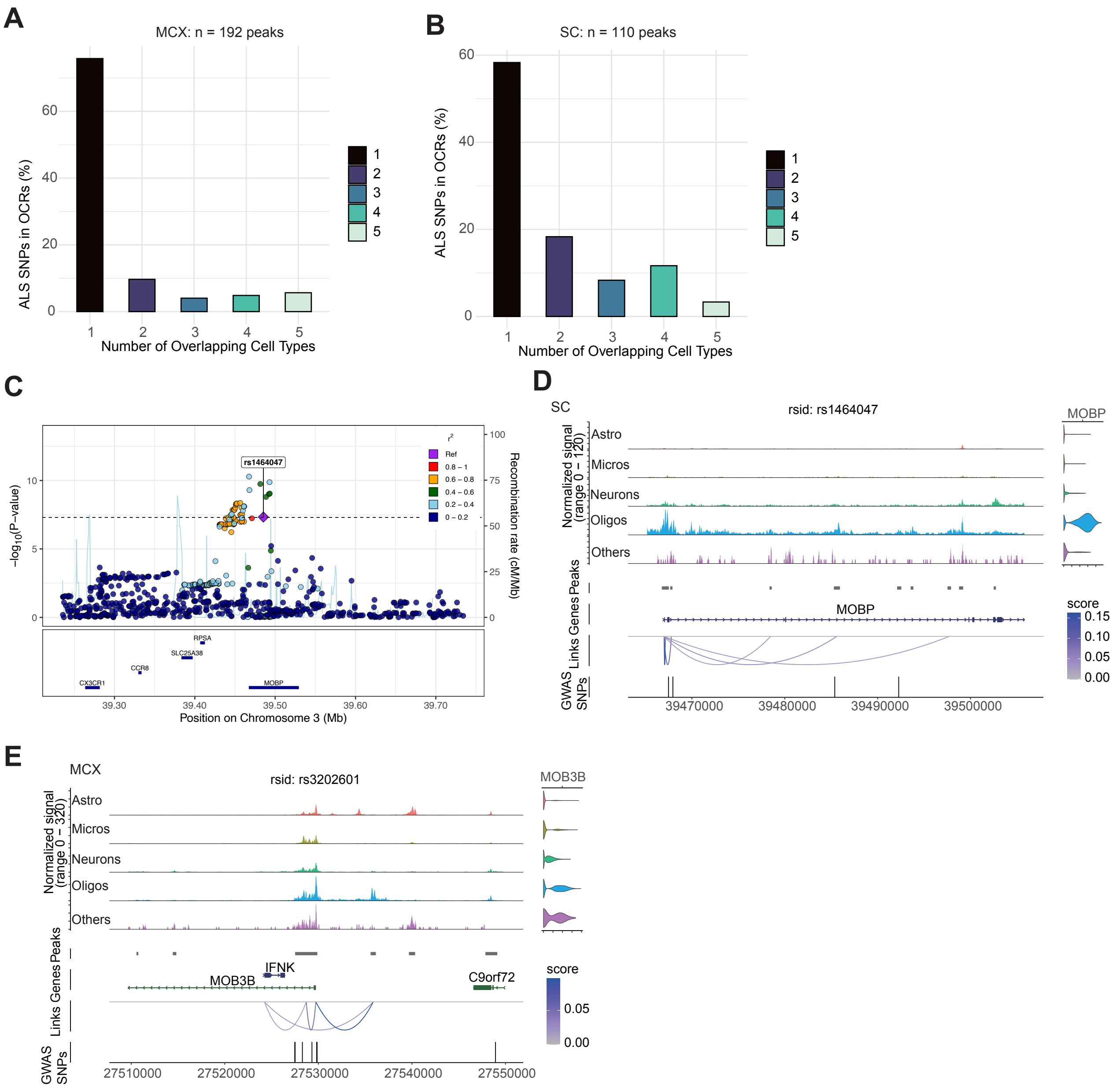

Supplementary Figure 8

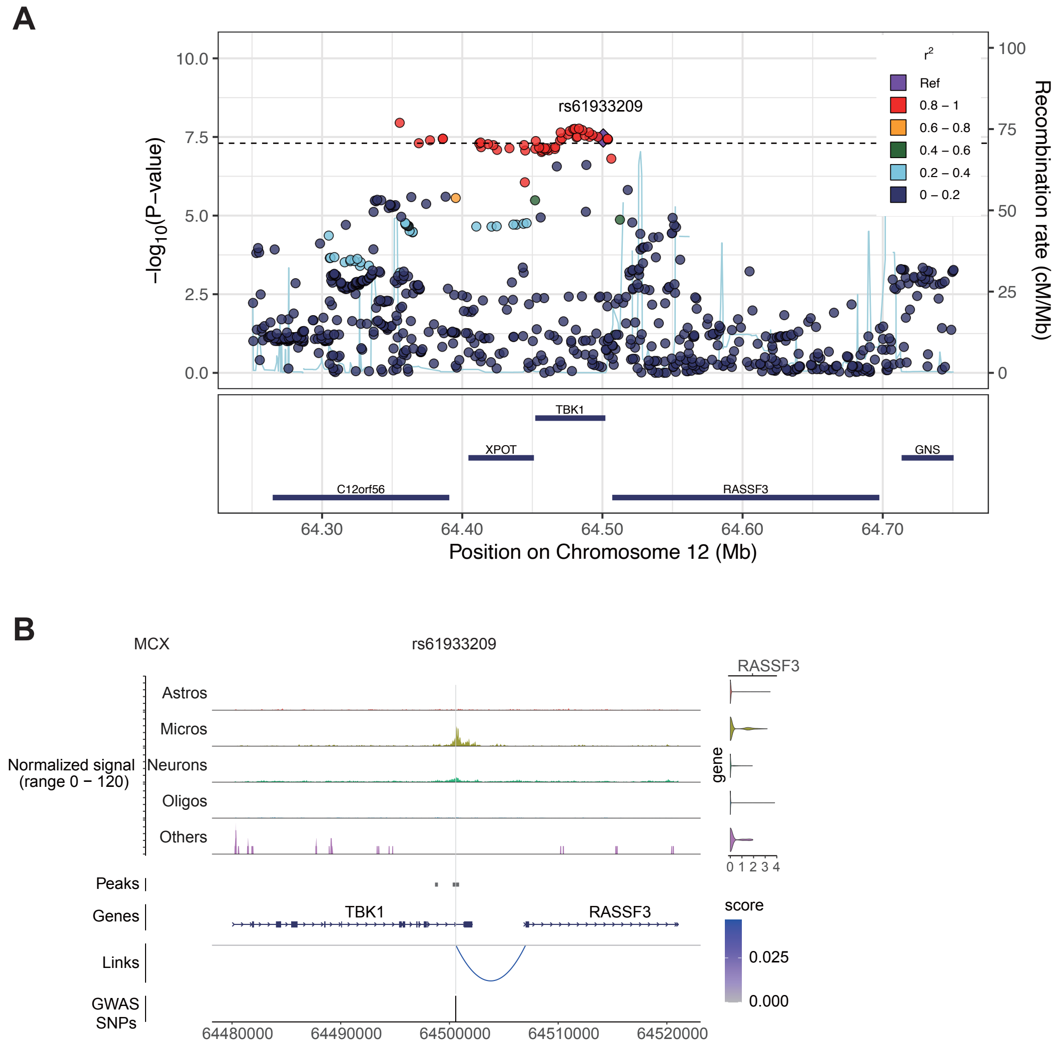

Supplementary Figure 9

Supplementary Figure 1 Characterization of the MCX and SC. (A) Distribution of unique molecular identifier counts and number of observed genes per sample in the motor cortex (MCX) (upper) and spinal cord (SC) (lower). **(B)** PCA plots of cell frequency and psudobulk RNA expression per sample in MCX and SC. The variations in PC1 and PC2 due to methodological differences were assessed using a two-tailed Welch's t-test. The corresponding p-values are indicated in the figure. **(C)** Number of nuclei retained after QC and filtrations per sample in the MCX (left) and SC (right). **(D)** Marker genes for neurons (left) and glial cells (right) in the MCX (upper panel) and SC (lower panel).

Supplementary Figure 2 Spatial Mapping of Cellular Populations in the Spinal Cord. (A) Spatial distribution of SC clusters in the SC of a human patient with amyotrophic lateral sclerosis (ALS). Cell frequencies were calculated using Cell2location.[54] **(B)** Annotated anatomical regions, as defined in Maniatis *et al.*[14] Multiple sections from the spinal cords were overlaid based on the anatomical clusters.

Supplementary Figure 3 Detailed information for cell–cell interactions. (A) Total number of significant cell-cell communications (CCCs) in ALS and healthy control (HC) subjects in the MCX and SC. **(B)** Interaction strength of CCC in the MCX and SC of ALS and HC groups. **(C)** Distribution of information flow in the MCX (left) and SC (right). The overall information flow of a signaling network is calculated by summarizing all communication probabilities in that network. The top signaling pathways colored in red were more enriched in ALS, and the bottom ones colored in blue were more enriched in the control. **(D)** Dot plot showing the changes in the expression of receptor categories involved in CCC across different cell populations. Dot size indicates statistical significance, while color represents the difference in module scores for each receptor group between ALS and control within each cell type. Red indicates higher scores in ALS. **(E)** Heatmaps showing differential sending signal strength (upper) and receiving signal strength (lower) in the MCX. Color represents the relative signaling strength of a signaling pathway across cell groups. The values are row-scaled.

Supplementary Figure 4 UMAP visualization of scDRS scores. The top panel shows the MCX, and the bottom panel shows the SC. The arrow indicates MNs in the SC.

Supplementary Figure 5 Gene expression patterns of ALS-related genes. (A, B) Dot plot showing the expression of ALS-related genes across cell types in the MCX **(A)** and SC **(B)**. **(C, D)** K-means clustering of ALS-related genes on PCA embeddings in the MCX **(C)** and SC **(D)**.

Supplementary Figure 6 Heatmaps depicting the number of significant genes identified at various adjusted P-value thresholds. **(A)** Results from the motor cortex (MCX); **(B)** results from the spinal cord (SC). Genes with positive log2 fold change are shown on the left side of each panel, while those with negative log2 fold change are shown on the right.

Supplementary Figure 7 snATACseq profiling. (A, B) Uniform manifold approximation and projection plot calculated based on a single-nucleus assay for transposase-accessible chromatin using sequencing (snATACseq) profiles in the MCX **(A)** and SC **(B)**. **(C, D)** Number of peaks and genomic distributions in MCX **(C)** and SC **(D)**. **(E, F**) UpSet plot showing the overlap of peaks among cell types in MCX **(E)** and SC **(F)**.

Supplementary Figure 8 Association of ALS-related SNPs and cell type-specific OCRs. (A, B) Distributions of the number of overlapping cell types for ALS-related SNPs in open chromatin regions (OCRs) in the MCX **(A)** and the SC **(B)**. **(C)** Locus plot of rs1464047 and adjacent loci. **(D, E)** Representative chromatin accessibility sequence tracks around candidate gene-regulatory SNPs located in an intron of MOBP in SC **(D)** and MOB3B in the MCX **(E)** and MOBP in the SC **(D)**.

Supplementary Figure 9 Gene-regulatory candidate SNPs with microglia-specific activation. **(A)** Locus plot of rs61933209 and adjacent loci. **(B)** Representative chromatin accessibility sequence track around candidate gene-regulatory SNPs in the MCX. rs61933209, located in an intron of TBK1, was significantly correlated with the gene expression of RASSF3. **(C)** Dot plot comparing the average RNA expression of *RASSF3* and *TBK1* in the MCX between ALS and control samples.
