# Supplementary material for: Single-nucleus multiome shows motor neuron glutamate overactivation in amyotrophic lateral sclerosis
Source: Brain. 2025 Nov 11;149(7):2480–94. doi: 10.1093/brain/awaf426 (PMC13337230; doi:10.1093/brain/awaf426)

Source Data

Page 2: GRM5 Western blot

Page 3: TDP-43 and GAPDH Western blot

(*Only the left two lanes are used on pages 2 and 3*)

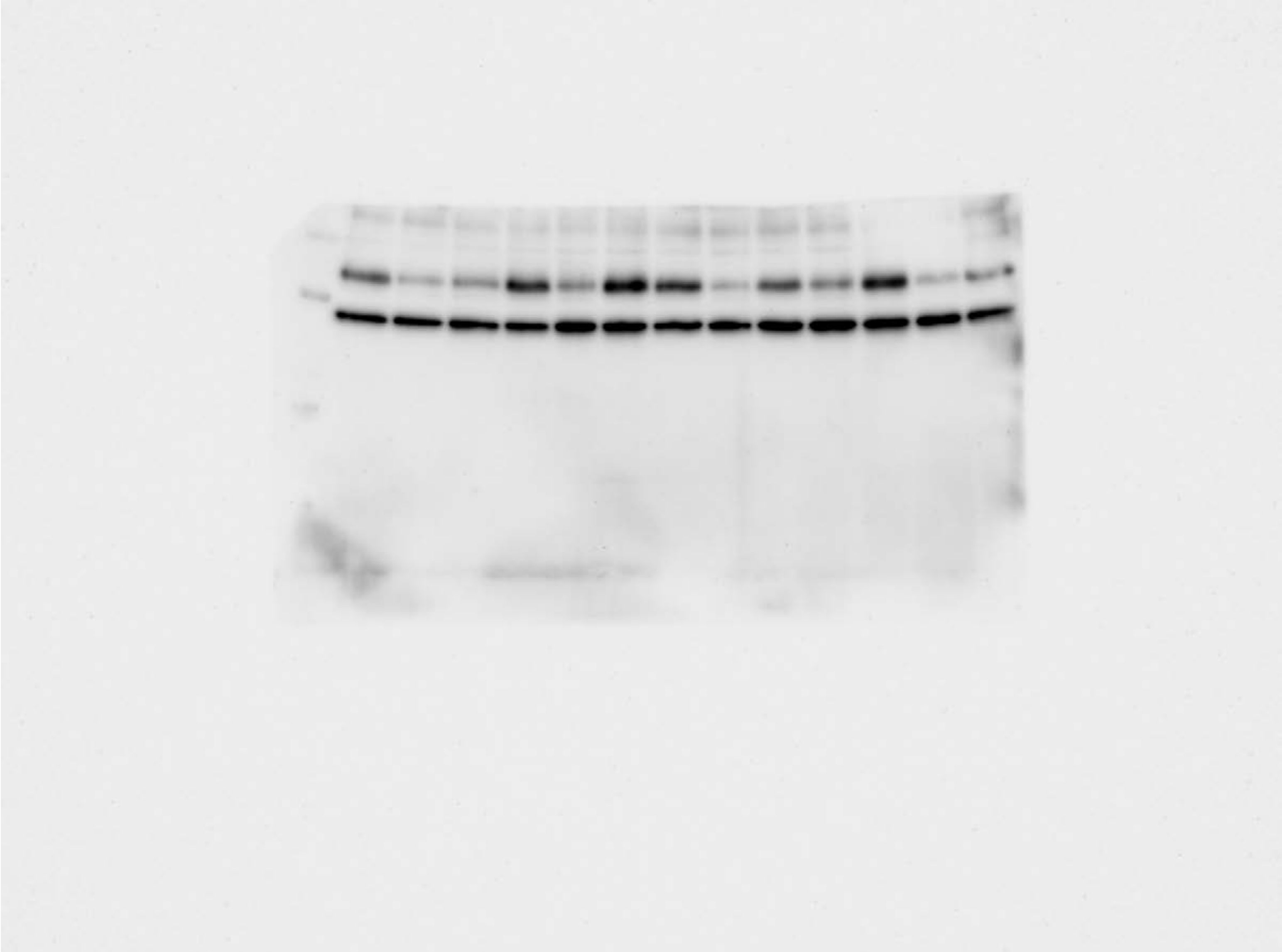

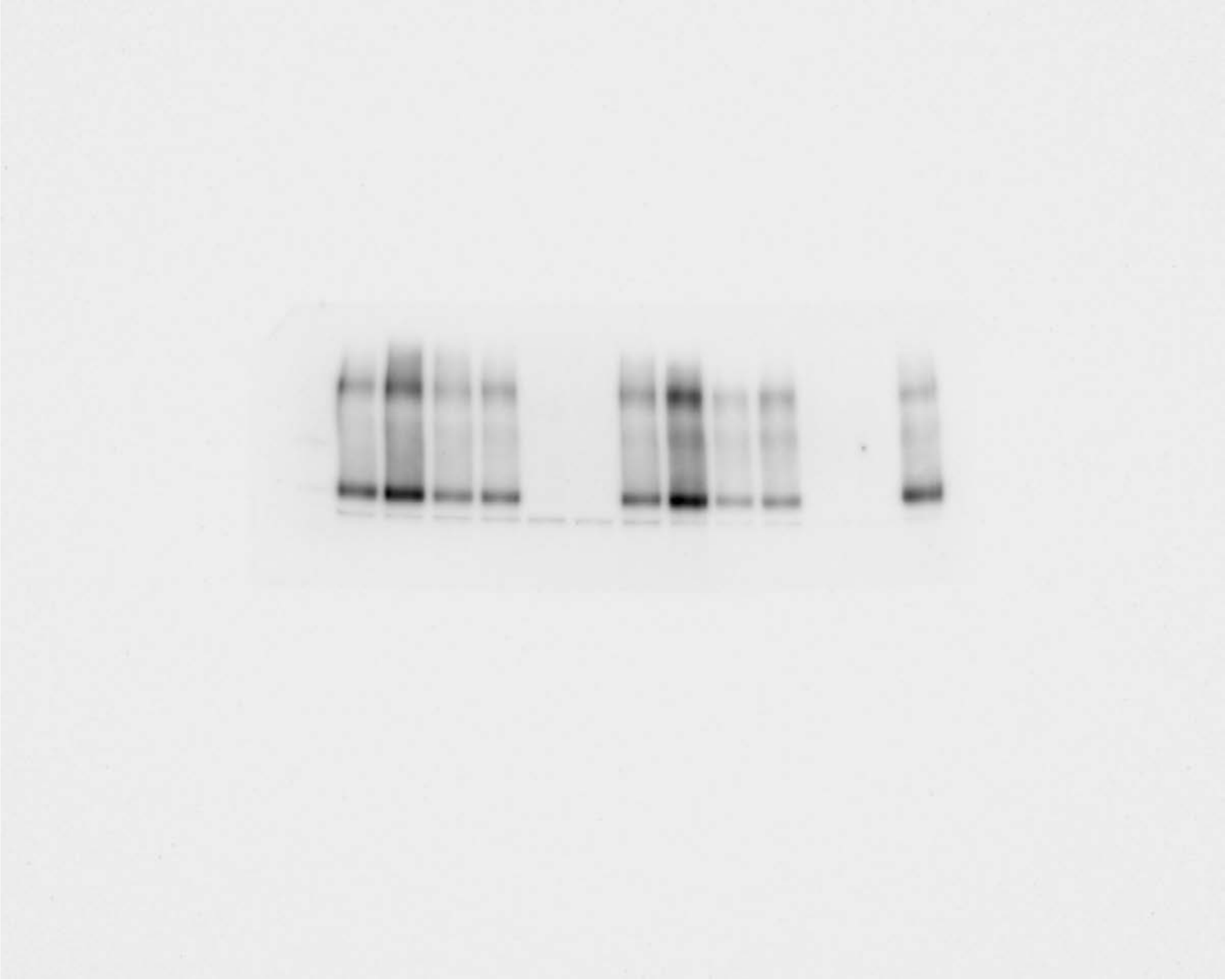

Supplement: awaf426_Supplementary_Data [file awaf426_supplementary_data.zip › brain-2025-00589-File010.pdf]
